# Overexpression of recombinant proteins containing non-canonical amino acids in *Vibrio natriegens*: p-azido-L-phenylalanine as coupling site for ^19^F-tags

**DOI:** 10.1007/s00726-022-03148-2

**Published:** 2022-04-13

**Authors:** Karina A. Stadler, Walter Becker, Barbara Darnhofer, Ruth Birner-Gruenberger, Klaus Zangger

**Affiliations:** 1 Institute of Chemistry, University of Graz, Heinrichstrasse 28, 8010 Graz, Austria; 2 Diagnostic and Research Institute of Pathology, Medical University of Graz, Stiftingtalstrasse 6, 8010 Graz, Austria; 3 Omics Center Graz, BioTechMed-Graz, Stiftingtalstrasse 24, 8010 Graz, Austria; 4 Institute of Chemical Technologies and Analytics, Faculty of Technical Chemistry, Technische Universität Wien, Getreidemarkt 9/164, 1060 Vienna, Austria; 5 Present Address: Department of Medical Biochemistry and Biophysics, Karolinska Institutet, Solnavägen 9, 17177 Stockholm, Sweden

**Keywords:** Unnatural amino acids, Orthogonal tRNA/tRNA synthetase, Stop codon suppression, ^19^F-NMR

## Abstract

*Vibrio natriegens* is the fastest growing organism identified so far. The minimum doubling time of only 9.4 min, the ability to utilize over 60 different carbon sources and its non-pathogenic properties make it an interesting alternative to *E. coli* as a new production host for recombinant proteins. We investigated the ability of the engineered *V. natriegens* strain, Vmax™ Express, to incorporate the non-canonical amino acid (ncAA) p-azido-L-phenylalanine (AzF) into recombinant proteins for NMR applications. AzF was incorporated into enhanced yellow fluorescent protein (EYFP) and MlaC, an intermembrane transport protein, by stop codon suppression. AzF incorporation into EYFP resulted in an improved suppression efficiency (SE) of up to 35.5 ± 0.8% and a protein titer of 26.7 ± 0.7 mg/L. The expression levels of MlaC-AzF even exceeded those of *E. coli* BL21 cells. For the recording of ^1^H-^15^N and ^19^F NMR spectra, EYFP-AzF was expressed and isotopically labeled in minimal medium and the newly introduced azido-group was used as coupling site for NMR sensitive ^19^F-tags. Our findings show that Vmax is a flexible expression host, suitable for the incorporation of ncAAs in recombinant proteins with the potential to surpass protein yields of *E. coli*. The presented method suggests the implementation of *V.* *natriegens* for expression of isotopically labeled proteins containing ncAAs, which can be chemically modified for the application in protein-observed ^19^F-NMR.

**Supplementary Information:**

The online version contains supplementary material available at 10.1007/s00726-022-03148-2.

## Introduction

*Vibrio natriegens* was already described in 1958 by William Payne (Payne 1958), but has only recently gained interest as a new production host for molecular biology, biotechnological processes and as cell-free protein synthesis system (Weinstock et al. 2016; Lee et al. 2019a; Hoff et al. 2020; Failmezger et al. 2018). With minimum doubling times of 9.4–9.8 min at optimal growth conditions, *V. natriegens* is the fastest growing organism isolated so far (Eagon 1962; Payne 1958; Thoma and Blombach 2021). This feature is resulting from the exceptional high number of ribosomes per cell (up to 115 000) and the organization of the genome on two chromosomes enabling parallel replications starting from two origins of replication (Aiyar et al. 2002; Lee et al. 2016; Wang et al. 2013; Maida et al. 2013). *V. natriegens* can grow to significantly higher cell densities compared to *Escherichia coli* (Hoff et al. 2020; Kormanová et al. 2020), it can tolerate a broad range of pH and it can use over 60 different carbon sources under aerobic conditions (Hoffart et al. 2017; Ellis et al. 2019; Tschirhart et al. 2019; Thoma and Blombach 2021). In contrast to other *Vibrio* species like *Vibrio* *cholerae*, *V. natriegens* is non-pathogenic and belongs to the biosafety level 1 (Thoma and Blombach 2021). In the field of biotechnology *V.* *natriegens* has already hit the headlines several times, being referred to as “next-generation workhorse” (Zhang et al. 2021), “next-generation whole-cell catalysis chassis” (Liu et al. 2022) or “host for rapid biotechnology” (Xu et al. 2021b). Native strains show the ability to grow on glycerol, galactose, rhamnose, maltose, fructose, mannitol or arabinose that also occur in by-products from industry or sustainable feedstocks. The growth rate on sucrose is even slightly higher compared to glucose (Hoffart et al. 2017; Ellis et al. 2019). Its rapid growth is directly connected to the substrate consumption rate (*qs*). On glucose minimal medium the *qs* is 3.9 g/(g × h) under aerobic and 7.8 g/(g × h) under anaerobic conditions and thereby two times higher compared to *E. coli*. *V.* *natriegens* has shown its applicability in bioreactors with different carbon sources under aerobic and anaerobic conditions in batch, fed-batch and high-cell-density cultivations (Hoffart et al. 2017; Thoma et al. 2021; Zhang et al. 2021; Liu et al. 2022; Erian et al. 2020).

Despite all these desirable features, so far *E.* *coli* still remains the most commonly used bacterial production host. However, the adaptation of methods from *E. coli* to *V.* *natriegens* is facilitated by several underlying metabolic and physiological similarities between these two strains. Both are gram-negative and facultatively anaerobic organisms. Their core carbon metabolism was found to be similar during aerobic growth on glucose minimal medium and both use the same pathways to synthesize canonical amino acids (Long et al. 2017). The genetic proximity to *E. coli* allows in many cases a direct transfer of *E. coli* optimized pET-plasmids into the engineered *V.* *natriegens* strain, Vmax™ Express (Synthetic Genomics 2017), that was equipped with a T7 RNA polymerase expression cassette (Xu et al. 2021a). Among others, Vmax is compatible with *lacUV5* and *ara*BAD promoters, frequently used antibiotic resistance cassettes including the ones for chloramphenicol, kanamycin, ampicillin and tetracycline, as well as commonly used origins of replication like pMB1, pUC, p15A and derivatives (Thoma and Blombach 2021; Hoff et al. 2020). Although *V.* *natriegens* is performing well with *E. coli* optimized plasmids, it is noteworthy that codon usage frequencies deviate up to two-fold between these species, thus codon optimization for *V. natriegens* is expected to improve protein production levels significantly (Hoff et al. 2020; Lee et al. 2016; Thoma and Blombach 2021).

However, to fully exploit the potential of *V.* *natriegens* as a production host, several methods need further optimization. The *in vivo* incorporation of non-canonical amino acids (ncAAs) in recombinant proteins is well-established in *E. coli*, yeast (Wang et al. 2006) and mammalian cells (Liu et al. 2007) but not in *V.* *natriegens*. It is a powerful tool in protein engineering to expand the genetic code that usually encodes the same 20 amino acids (Drienovská and Roelfes 2020; Ryu and Schultz 2006; Young et al. 2010), except for pyrrolysine (Srinivasan et al. 2002) and selenocysteine (Böck et al. 1991). Specific ncAAs can be incorporated into specific sites of the protein by creating new codons or reprogramming existing ones. This method, called stop codon suppression, relies on the use of an orthogonal tRNA/aminoacyl-tRNA synthetase pair. The orthogonal tRNA must not be recognized by the endogenous tRNA synthetase. It delivers the ncAA in response to a unique codon that does not encode any of the 20 natural amino acids. The orthogonal tRNA synthetase must aminoacylate the tRNA with only the desired ncAA but no endogenous amino acids (Wang et al. 2006). Stop codon suppression can be used for the incorporation of site-specific post-translational modifications, biophysical probes or labels (Gan et al. 2016). The amber stop codon UAG is used the least in *E. coli* (Drienovská and Roelfes 2020) and thus commonly assigned to the desired ncAA, while the tRNA anticodon is mutated to CUA. Only recently, *V. natriegens* was used for the incorporation of propargyl-L-lysine into green fluorescent protein (GFP) as the first attempt of ncAA incorporation (Ozer and Alfonta 2021). Unfortunately, the results show only 0.5 ± 0.0% suppression efficiency (SE), the relative amount of protein of the mutant (MT) compared to the wildtype (WT).

**Scheme 1:**
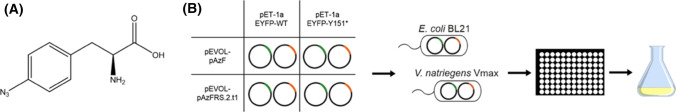
**A** Structure of p-azido-L-phenylalanine. **B** Work flow for EYFP expression: Co-transformation of plasmids into *E. coli* BL21(DE3) and *V. natriegens* Vmax™ Express cells, selection of variants by a 96-well-plate screening, shaking flask expressions with best variants

In this work, we demonstrate SE of up to 35.5 ± 0.8% obtained in Vmax. Plasmids designed for and commonly used in *E. coli* were transferred to the engineered *V. natriegens* strain Vmax™ Express for successful expression of recombinant proteins containing p-azido-L-phenylalanine (AzF) (Scheme 1A). Additionally, the newly introduced azido-group was used as coupling site for ^19^F-containing tags, applicable in NMR studies.

## Results and discussion

Enhanced yellow fluorescence protein (EYFP) (FPbase 2021) was chosen as model protein due to its fluorescent properties. EYFP was expressed with a Z-domain (Z2) tag, that was cleaved off by tobacco etch virus (TEV) protease after expression. The mutation of the surface residue Y151 to an amber stop codon (TAG) was stated in literature to not affect the fluorophore, protein stability or correct folding (Young et al. 2010; Gan et al. 2016; Wang et al. 2010). The intermembrane phospholipid transport system binding protein, MlaC, was expressed with a N-utilization substance (NusA) tag that was cleaved off by TEV protease after expression. The position K100, a surface residue in a loop, was selected as incorporation site for AzF and was therefore mutated to an amber stop codon. The incorporation site was suspected to be easily accessible by chemicals and to not affect correct folding. Incorporation sites were mutated by site directed mutagenesis (Supporting Information, Table S1).

The orthogonal pair consisting of the tyrosyl-tRNA synthetase and the corresponding suppressor tRNA originating from *Methanocaldococcus jannaschii* has been proven to be successful in *E. coli* and was therefore selected for this study. This system has been used for incorporation of more than 30 ncAAs as fluorescent, photo crosslinking or metal ion binding tags or probes for nuclear magnetic resonance (NMR), IR or crystallography (Young et al. 2010; Chin et al. 2002).

We used pEVOL plasmids with inserts for different variants of the *M. jannaschii* AzF-tRNA synthetase, pEVOL-pAzF and pEVOL-pAzFRS.2.t1 (Supporting Information, Table S2). pEVOL-pAzF was optimized for the incorporation of AzF in *E. coli* in response to an amber stop codon, while the pAzFRS.2.t1 plasmid can additionally incorporate several other ncAAs. Both plasmids carry a modified suppressor tRNA, tRNA^opt^_CUA_, that showed increased ncAA incorporation efficiency (Young et al. 2010; Amiram et al. 2015). *V.* *natriegens* Vmax and *E. coli* BL21(DE3) cells were transformed with a pET plasmid coding for the WT- or MT-protein and a pEVOL plasmid, respectively (Scheme 1B).

For the expression and incorporation of AzF in BL21 cells, the optimized procedure from Young et al. (2010) was used. Thus, BL21 cells were grown in 1xM9 minimal medium to late growth phase (~ OD_600_ 1.4) before pET-1a and pEVOL plasmids were induced with IPTG and arabinose, respectively. It is noteworthy that *V.* *natriegens* is a halophilic bacterium that requires Na^+^ ions for its cell proliferation. Under sodium ion limiting conditions, it retains metabolic activity but growth cannot be sustained (Webb and Payne 1971; Thoma and Blombach 2021). Most cultivation media are supplemented with 15 g/L NaCl (257 mM Na^+^) (Thoma and Blombach 2021). In our study, we used the medium optimization procedure described by Becker et al. (2019), where M9 minimal medium was supplemented with different concentrations of Na_2_HPO_4_/KH_2_PO_4_ buffer and NaCl. Both proteins were expressed in M9 minimal medium containing 1x, 3x, 6x and 8x concentrated NaCl and buffer components (Supporting Information, Table S3 + S4). Thereby, a total range of 110–880 mM Na^+^ was tested for best protein expression yields. For EYFP and MlaC the best conditions were found to be with M9 minimal medium containing 3x concentrated NaCl and buffer components (3xM9) corresponding to 330 mM Na^+^ (Supporting Information, Fig. S1).

The timepoint for induction in Vmax is independent of the growth phase (Becker et al. 2019), thus pEVOL plasmids were induced 30 min after inoculation to allow expression of the orthogonal pair prior to EYFP expression.

### EYFP expression

EYFP was used for a 96-well-plate-based expression screening in which tRNA synthetase variants were tested for their compatibility with the Vmax expression system. The best variants were selected for expression in shaking flasks (Scheme 1B). The mutant EYFP-Y151* was expressed with AzF for incorporation (EYFP-Y151AzF) and without AzF to monitor background fluorescence (EYFP-Y151*). EYFP-WT was expressed as positive control.

**Fig. 1:**
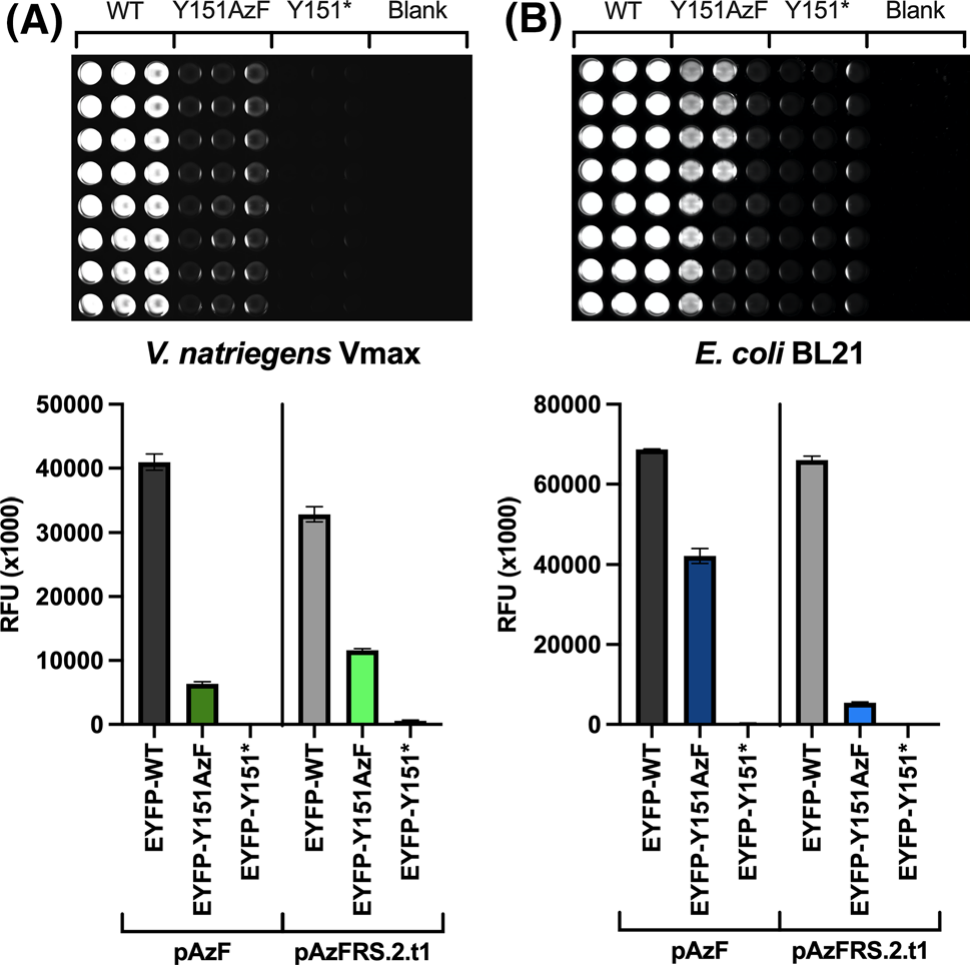
96-well-plate expression in **A** *V.* *natriegens* Vmax and **B** *E. coli* BL21. Fluorescence data were obtained from EYFP-WT (positive control), EYFP-Y151AzF (MT with incorporated AzF), EYFP-Y151* without addition of AzF (MT without AzF/negative control) and Blanks. Fluorescence data are normalized by OD_600_ and shown as mean ± standard deviation (STD) calculated from *n* = 3 independently grown cultures (*p* < 0.0001)

The results show that Vmax can use both tRNA synthetase variants to incorporate AzF, even though the expression level is significantly lower compared to the WT (Fig. 1A). The SE calculated from the relative fluorescence units (RFU) was 15.5 ± 0.8% for pEVOL-pAzF and 35.5 ± 0.8% for pEVOL-pAzFRS.2.t1. In BL21 cells the SE was 61.4 ± 2.7% for pEVOL-pAzF, while with the pEVOL-pAzFRS.2.t1 variant only 8.2 ± 0.3% could be obtained (Fig. 1B). Noteworthy, Vmax could obtain a better SE with the pEVOL-pAzFRS.2.t1 variant even though the plasmid was codon-optimized for *E. coli*. The negative controls show low background fluorescence of 0.5 ± 0.0% for BL21 and ≤ 1.8 ± 0.3% for Vmax, which confirms that no incorporation is taking place if AzF is missing, consequently the full-length protein cannot be expressed. However, the expression levels of WT proteins were better in BL21 compared to Vmax. Considering that BL21 cells were grown to an OD_600_ of 1.4 before being transferred to 96-well-plates, while Vmax cells were grown directly in the plates, the lower expression in Vmax might be due to suboptimal growth conditions (insufficient shaking, reduced aeration) in the 96-well-plates. All data and the pipetting scheme are provided in Supporting Information (Fig. S2 and Table S5).

For further studies, the best variants were selected for each strain and expressions were performed in shaking flasks, followed by a protein purification procedure. pEVOL-pAzFRS.2.t1 was selected for further expression experiments in Vmax, while for BL21 the pEVOL-pAzF variant was used.

**Fig. 2:**
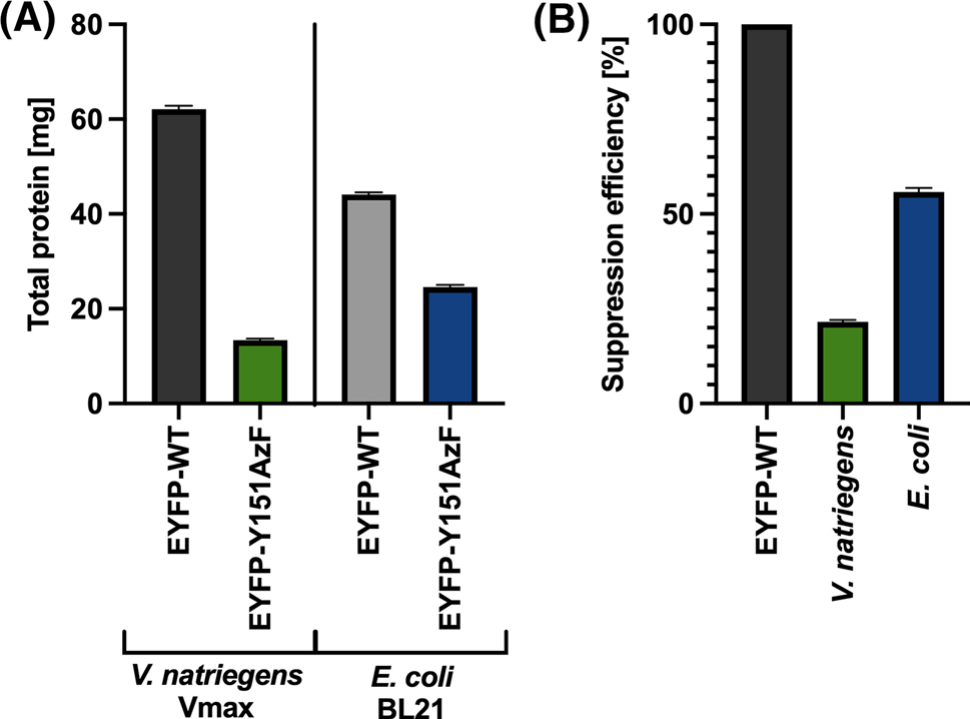
Total protein amount of EYFP determined by BCA assay. **A** Total protein amount of EYFP-WT and EYFP-Y151AzF expressed in Vmax and BL21. **B** SE calculated from total protein amounts after purification. Data are shown as mean ± STD calculated from *n* = 3 measuring repeats (*p* < 0.0001)

Proteins were expressed in 500 mL M9 minimal medium containing ^15^N-ammonium chloride and ^13^C-glucose for isotopic labeling as needed for NMR studies. After protein purification, the total protein amount (Fig. 2A) and the specific protein amount were determined (Supporting Information, Table S6-S7). SE was calculated from total protein results (Fig. 2B): In Vmax 13.4 ± 0.3 mg EYFP-Y151AzF resulted in a SE of 21.5 ± 0.5%, while in BL21 the total protein amount was 24.6 ± 0.5 mg with a SE of 55.8 ± 1.1%. Similar results were obtained from specific protein determination. The protein amount resulting from EYFP-WT is higher in Vmax compared to BL21, while the expression of EYFP-Y151AzF gives better results in BL21. Additional expression details are given in the Supporting Information (Table S8).

The SE of ~ 56% we obtained in BL21 cells is compatible with the findings of a similar study that were published in the context of the first pEVOL generation (Young et al. 2010). Young states a maximum yield of > 50% under optimal conditions for the incorporation of p-acetyl-phenylalanine into GFP at position Y151. For the incorporation of AzF an average yield of > 35% relative to GFP-WT was indicated. However, in contrary to most other studies, we used minimal medium for protein expression, which makes a direct comparison to previous results difficult. As *V.* *natriegens* is a new candidate in the field of genetic code expansion, very few data exist about incorporation of ncAAs. The first attempt was successful but yielded in only 0.5% SE resulting in ~ 1 mg protein/L medium (Ozer and Alfonta 2021). More promising, our findings show that Vmax can reach much higher yields. The expression of EYFP-Y151AzF on minimal medium yielded in ~ 27 mg/L. Compared to this study, AzF incorporation into EYFP resulted in a 71-fold increased SE of up to 35.5 ± 0.8%.

Naturally, the availability of orthogonal aminoacyl-tRNA synthetase/suppressor tRNA pairs is crucial for this method. Although pEVOL plasmids were successfully used in Vmax, a detailed screening of mutants that obtain better results in *V.* *natriegens* might not only help to improve the performance, but also to better understand the similarities and differences compared to the translational system of *E. coli*.

**Fig. 3:**
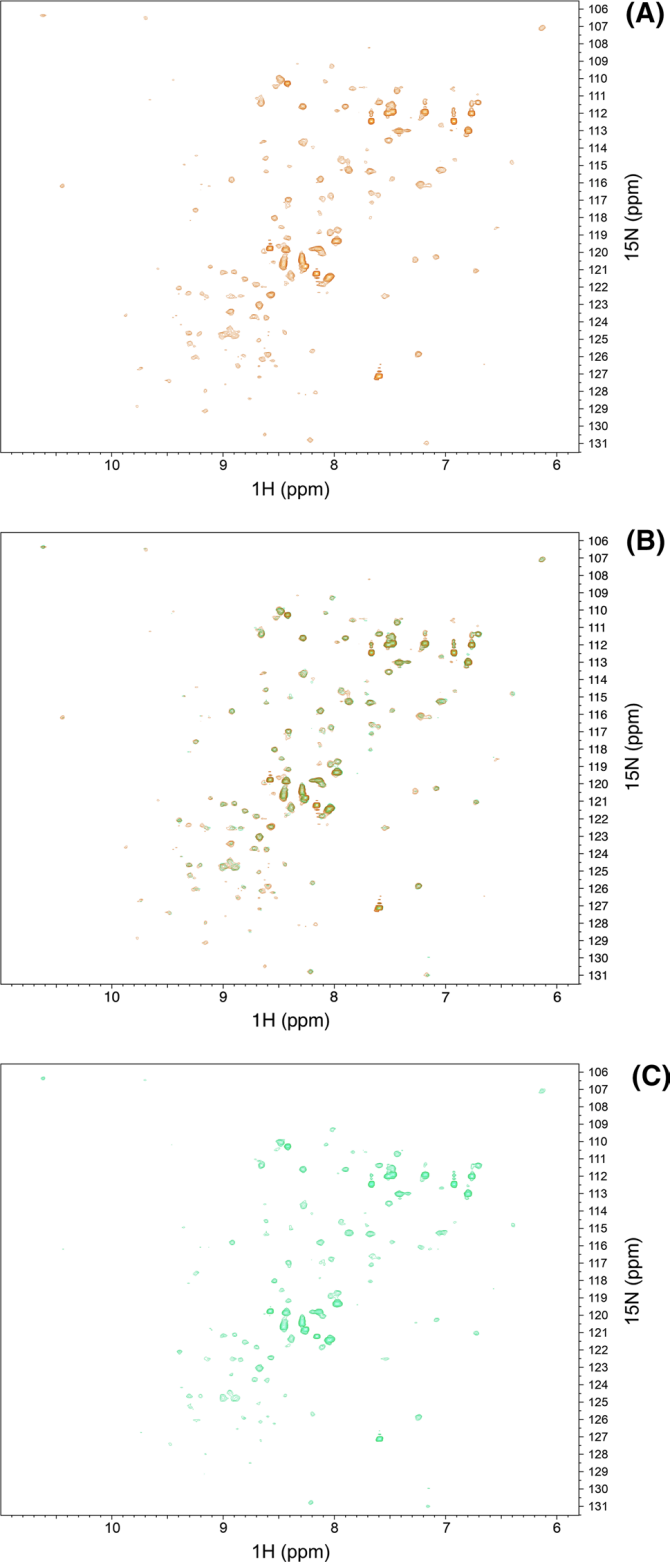
^1^H-^15^N HSQC spectra of EYFP-Y151AzF. **A** EYFP-Y151AzF expressed in *V.* *natriegens* Vmax. **B** Overlap of EYFP-Y151AzF spectra. **C** EYFP-Y151AzF expressed in *E. coli* BL21

As the process of ncAA incorporation may eventually lead to unfolded, misfolded or not fully translated proteins it is necessary to check if the expressed protein is correctly translated and folded (Drienovská et al. 2020). Circular dichroism or fluorescence based assays can be used to obtain structural information or detect changes in the chemical environment (Kessenbrock and Groth 2017). However, the recording of ^1^H-^15^N Heteronuclear Single Quantum Coherence (HSQC) spectra is not only quick and easy, it is also a highly sensitive probe for protein structure, since the resonance frequencies are very responsive to even small changes in the local environment of a protein (Fig. 3). Each amino acid produces a peak from the ^1^H-^15^N pair in the amide group (except proline and N-terminal amino acid). Consequently, it gives an overview over the backbone and thus the folding of the protein with atomic resolution (Becker et al. 2018). The HSQC spectrum of EYFP-Y151AzF expressed in Vmax (Fig. 3A) shows the same ^1^H-^15^N correlations as EYFP-Y151AzF expressed in BL21 (Fig. 3C), which results in an identical overlap of both spectra (Fig. 3B).

To determine whether or not AzF was incorporated at position Y151, all proteins were analysed by mass spectrometry measurements. Monoisotopic masses of peptide digest fragments showed no modifications in EYFP-WT, while the AzF modification could be found in all fragments originating from EYFP-Y151AzF. Additionally, AzF incorporation (resulting in a mass difference of 25 Da) was confirmed by intact mass measurements (Supporting Information, Table S9-S10).

### MlaC expression

**Fig. 4:**
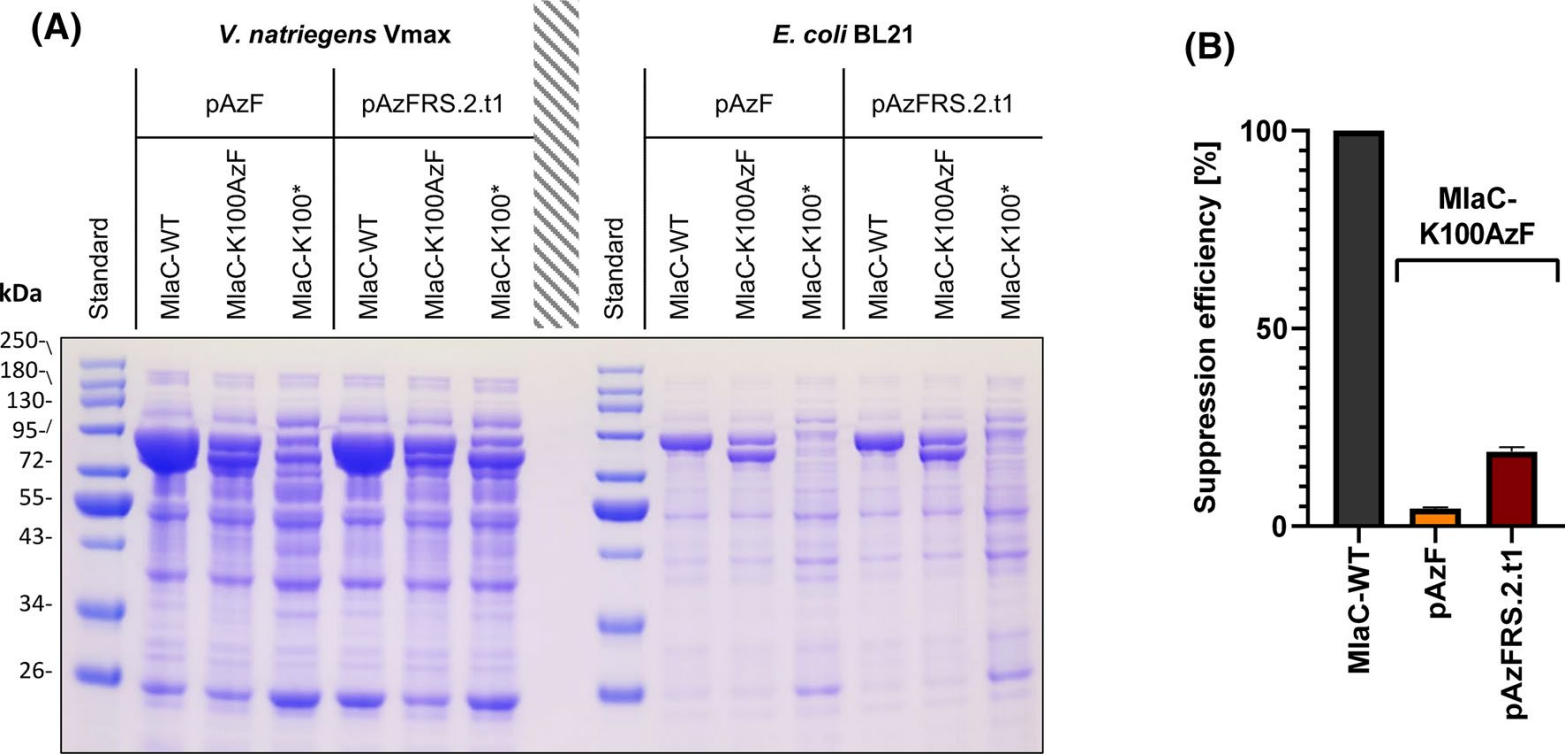
**A** SDS-PAGE of MlaC-WT (positive control), MlaC-K100AzF (MT with incorporated AzF) and MlaC-K100* (MT without AzF/negative control) expressed in Vmax and BL21 cells with pEVOL-plasmids carrying the pAzF or pAzFRS.2.t1 tRNA synthetase variant. **B** SE of MlaC-K100AzF expressed with pAzF and pAzFRS.2.t1 tRNA synthetase variant, calculated from specific protein concentration. MlaC-K100AzF data are shown as mean ± STD calculated from *n* = 3 measuring repeats (*p* < 0.0001)

In the MlaC protein sequence, the position K100 was chosen as incorporation site. As described before, the mutant was expressed with and without the addition of AzF and MlaC-WT was expressed as positive control (no isotopic labeling). Cell lysate supernatants from 25 mL cultures in shaking flasks were separated by SDS-PAGE. The protein bands of full-length MlaC (6xHis-NusA-MlaC: 78.8 kDa) and fragment MlaC (6xHis-NusA-MlaC-K100*: 69.0 kDa) appear between the 72 and 95 kDa-standard (proteins may appear bigger due to sterical effects). These results show an increased expression for both tRNA synthetase variants in Vmax compared to BL21 (Fig. 4A). However, it is noticeable that for both strains the protein band that represents full-length MlaC-K100AzF is only slightly stronger than the band of fragment MlaC. By those means, approximately half of the protein gets lost by incomplete incorporation of the ncAA. A limitation frequently occurring in stop codon suppression, is the competition between the endogenous release factor 1 (RF1) and the orthogonal tRNA for the UAG codon. This often leads to premature termination and thereby reduces the yield of full-length protein significantly (Amiram et al. 2015).

Shaking flask expressions of MlaC in Vmax were done in 250 mL cultures. The determination of specific protein concentrations resulted in 14.5 ± 0.2 mg protein for the WT, 0.7 ± 0.0 mg for MlaC-K100AzF pAzF and 2.7 ± 0.2 mg for MlaC-K100AzF pAzFRS.2.t1. These values resulted in SE of 4.5 ± 0.3% and 18.8 ± 1.1%, respectively (Fig. 4B and Supporting Information, Table S11-S12).

For MlaC-WT no modifications could be found in tryptic and chymotryptic fragments. The incorporation of AzF was successfully confirmed for MlaC-K100AzF of both variants by detection of chymotryptic fragments. However, the trypsin digest revealed both, modified and unmodified fragments, indicating that lysine is still present at position K100 in some proteins. Considering the high specificity of pEVOL-plasmids for AzF, a carry-over of WT protein during purification is more probable than the incorporation of lysine by the orthogonal tRNA synthetase (Supporting Information, Table S13).

Summed up, the expression levels surpassed those of *E. coli* and the best variant produced a titer of ~ 11 mg/L MlaC-K100AzF. Of course, the incorporation efficiency and thus the protein yield is dependent on various factors like the type of protein, incorporation site, incorporation method, type of non-canonical amino acid, strain specific codon usage, the orthogonal pair and its compatibility with the endogenous translation machinery (Young et al. 2010). Considering that none of these factors was specifically designed or optimized for *V.* *natriegens*, our results point out the flexibility and the enormous potential of this new production host.

### Coupling of ^19^F-tags

As the most prominent orthogonal pairs are derived from tyrosine and pyrrolysine (Drienovská and Roelfes 2020), it is often difficult and time-consuming to alter the binding pocket in the orthogonal tRNA synthetase to specifically bind ncAAs that differ substantially in structure (Bryson et al. 2017). Hence, the coupling of functional molecules to an azido-ncAA by copper-catalyzed azide-alkyne cycloaddition (CuAAC) is a way to circumvent this issue. CuAAC is well-known for its high specificity and reaction speed under mild reaction conditions and is therefore commonly used for the coupling of analytical probes or fluorescent molecules for cell imaging (Lee et al. 2019b; He et al. 2016).

In our study, we used CuAAC for the coupling of NMR sensitive ^19^F-labelled molecules. The demand for sensitive NMR probes is especially high in protein NMR where high molecular weights lead to signal reduction and signal overlap. The properties of ^19^F-tags can help to tackle these challenges in protein studies. ^19^F nuclei are hyperresponsive to changes in the chemical environment, which makes them a useful probe for studying conformational dynamics, protein folding, protein ligand interactions and corresponding structural effects (Arntson and Pomerantz 2016; Becker et al. 2018).

**Fig. 5:**
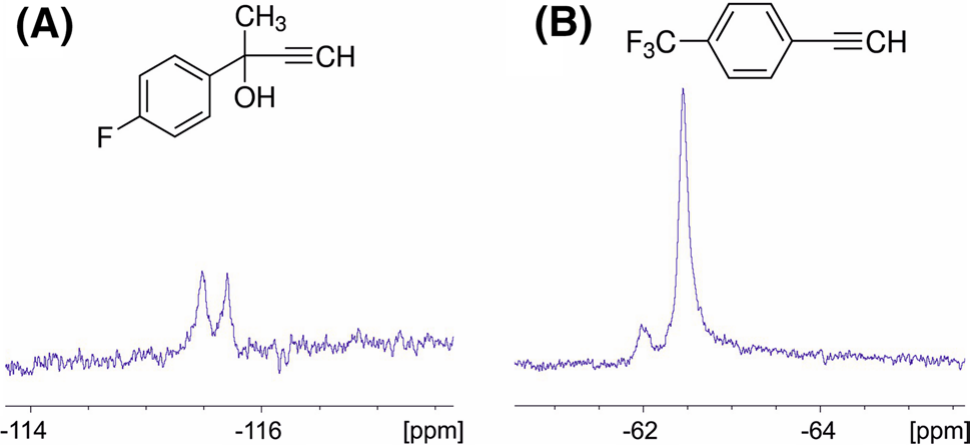
Chemical structure of ^19^F-tags and ^19^F-NMR spectra of EYFP-Y151AzF after copper-catalyzed azide-alkyne cycloaddition of **A** 2-(4-Fluorphenyl)-3-butin-2-ol or **B** 4-Ethinyl-α,α,α-trifluortoluol

We used the newly introduced azido-group of EYFP-Y151AzF as coupling site for ^19^F-tags. Two different alkyne tags, namely 2-(4-fluorphenyl)-3-butin-2-ol (^19^F-tag A) and 4-ethinyl-α,α,α-trifluortoluol (^19^F-tag B) were coupled to the azido-group by CuAAC in a 1 h-reaction-procedure (Fig. 5). The reaction mixture was then dialysed over night against NMR buffer and ^19^F-NMR spectra were recorded. For each of the samples, the ^19^F spectrum shows two peaks, of which one is the signal of the successfully coupled ^19^F-tag. The second signal shows unbound ^19^F-tag molecules that are still present in the sample. Spectra of pure tags were recorded for comparison. The full ^19^F-NMR spectra and spectra of pure tags are available in the Supporting Information (Figs. S3–S6). Additionally, the intact mass was measured to confirm the coupling reaction and determine the coupling efficiency. These results confirm that 13.5% of EYFP-Y151AzF were successfully coupled to ^19^F-tag A, while ^19^F-tag B was covalently coupled to 20.8% of azido-groups in a separate reaction (Supporting Information, Table S14).

Even the moderate coupling efficiency of 14–21%, results in sufficient high ^19^F-signals to monitor environmental changes of the protein. Consequently, the here presented method can be applied for protein-observed ^19^F NMR. This method is on the rise as a tool for drug discovery and optimization (Divakaran et al. 2019). The binding of ligands changes the chemical environment of ^19^F labeled proteins, which causes ^19^F-frequencies to shift. At the same time nonspecific binding events can be differentiated. Thereby, new drug candidates and corresponding binding sites and affinities can be identified (Buchholz and Pomerantz 2021).

## Conclusions

Although *E. coli* has its well-deserved position as the most popular bacterial protein expression host, a look beyond the existing systems brought up a highly interesting alternative. *Vibrio natriegens* can help to speed up lab work with faster growth rates and a flexible induction time point (Becker et al. 2019). While up to date *E. coli* still remains the first-to-try protein expression host, the direct transfer of plasmids into *V.* *natriegens* is time and cost saving and allows the implementation of a flexible toolbox of plasmids that can be used for both production hosts. According to a recent study by Xu et al. (2021a), the chance to obtain higher protein yields in *V.* *natriegens* from direct transfer of pET-plasmids is at least 10%. The additional codon-optimization of plasmids for *V.* *natriegens* will result in even higher yields.

Especially for NMR, protein yields need to be sufficiently high to obtain a reasonable signal-to-noise ratio. Triple resonance experiments require isotopic labeling, consequently, cells need to grow on minimal medium supplemented by isotopically labeled compounds. Vmax was so far successfully used for the expression of isotopically labeled FK506-binding protein (FKBP) and EYFP. In prior studies, we reported equally high expression levels of Vmax among non-, single (^15^N)-, and double-labeled (^15^N/^13^C) proteins (Becker et al. 2019). For future studies, also simultaneous labeling of proteins with ^15^N, ^13^C and ^2^H should be considered. Some NMR experiments require additional ^2^H-labeling; hence cells need to grow on deuterated medium. As this often leads to reduced protein yields, it might be interesting to elucidate the performance of Vmax cells in triple-labeling minimal medium (Venditti et al. 2012).

The method of ncAA incorporation and subsequent modification was applied in the past with great success in *E. coli* BL21(DE3) cells. Lindstedt et al. (Lindstedt et al. 2021) reports improved activity of a single-domain antibody against Alzheimer’s after incorporation of dehydroalanine into the CDR3 loop and systematic activity maturation by further chemical modifications. Another study presents a new artificial enzyme that was constructed by incorporation of p-amino-phenylalanine into LmrR, a lactococcal multidrug resistance regulator. The hydrazone forming reaction was fine-tuned by a directed evolution approach. The introduction of the catalytic ncAA and subsequent mutations of amino acids in or near the active site resulted in a 100-fold higher turnover frequency compared to the initial variant. Hence, the authors are suggesting the combination of genetic code expansion and directed evolution for the design of new-to-nature enzymes. (Drienovská and Roelfes 2020; Mayer et al. 2019). These and other studies, highlight the importance of ncAA incorporation as powerful protein engineering method. Applying and optimizing ncAA incorporation in *V.* *natriegens* will help to boost the implementation of this new expression host in the labs.

In this study, we present for the first time the expression of isotopically labeled proteins containing ncAAs for the application in NMR studies. Compared to a prior study in *V.* *natriegens*, suppression efficiency was increased 71-fold. Upon chemical coupling of ^19^F-tags, the AzF mutant proteins can be used for protein-observed ^19^F NMR. The combined approach with ^1^H-^15^N and ^19^F NMR can be used to identify ligand binding sites with amino acid resolution. Thus, more efforts should be made to fully exploit the potential of *V. natriegens* and further explore performance and limitations of ncAA incorporation. Future studies should be extended to numerous other proteins at different incorporation sites with different ncAA using different orthogonal pairs.

Unfortunately, we are still lagging behind in terms of detailed understanding of this organism. As research around *V.* *natriegens* is speeding up, some limitations we were facing at the beginning of this study, have been addressed in the meantime. By now, the construction of plasmids got facilitated as the Marburg collection offers a flexible collection with 191 genetic parts optimized specifically for *V.* *natriegens* (Stukenberg et al. 2021). However, the metabolism, its regulation and the translational machinery are still bearing a lot of knowledge gaps. The same is true for its most prominent feature, the rapid growth rate (Thoma and Blombach 2021; Hoff et al. 2020). *V.* *natriegens* is gaining interest in research groups across the globe as host for protein expression and metabolic engineering. Thus, we need a thorough investigation of these mechanisms to create a solid base for further strain optimization and finetuning of methods.

## Materials and methods

### Chemicals

All reagents were purchased in analytical grade. p-Azido-L-phenylalanine ≥ 98% (CAS 33173-53-4) was purchased from Santa Cruz Bioscience. Ammonium chloride (15N, 99%) 98% (CAS 39466-62-1) was purchased from Eurisotop. D-Glucose (U-13C6, 99%) 98% (CAS 110187-42-3) was purchased from Cambridge Isotope Laboratories, Inc. Tris(3-hydroxypropyl-triazolylmethyl)amine 95% (CAS 760952-88-3), aminoguanidin-hydrochlorid ≥ 98% (CAS 1937-19-5), 2-(4-fluorphenyl)-3-butin-2-ol 90% (CAS 159028-51-0) and 4-ethinyl-α,α,α-trifluortoluol 97% (CAS 705-31-7) were purchased from Sigma-Aldrich.

### Bacterial strains and plasmids

All experiments were performed with *V. natriegens* strain Vmax™ Express (Synthetic Genomics 2017) and *E. coli* BL21(DE3) (Novagen®).

The pET-1a vector was derived from pETM-13 (Stier 2021) (EMBL Heidelberg) with a pBR322 ori, a kanamycin resistance gene (KanR) and either a 6xHis-Z2-TEV-EYFP or a 6xHis-NusA-TEV-MlaC insert (Supporting Information, Table S1) under the control of the T7/*lac*-operon. pEVOL-pAzF (Chin et al. 2002) (Addgene #31,186) and pEVOL-pAZFRS.2.t1 (Amiram et al. 2015) (Addgene #73546; GenBank ID KT996140) plasmids carry a p15A ori and encode the chloramphenicol resistance gene (CmR) and optimized *M. jannaschii* tRNA synthetase and tRNA under the control of the *ara*BAD operon.

### Preparation of electrocompetent cells

#### *V. natriegens* Vmax

10 mL enhanced 2xYT medium (32 g/L tryptone, 20 g/L yeast extract, 17 g/L NaCl, 0.2% glucose, 17.6 mM Na_2_HPO_4_, pH 7.4) was inoculated with Vmax Express cells and incubated at 30 °C and 200 rpm over night. 150 mL enhanced 2xYT medium was inoculated with the over night-culture (ONC) to an OD_600_ of 0.05 and incubated in a shaking flask at 30 °C and 170 rpm to a final OD_600_ of 0.5 (approx. 1.5 h). The culture was then centrifuged at 3900×*g* (Sigma 6-16 K, 12,165-H) for 20 min at 4 °C. The pellet was washed with electroporation buffer (680 mM sucrose, 7 mM K_2_HPO_4_, pH 7) and centrifuged again (3900×*g*, 15 min, 4 °C). The wash was repeated three times to a total of four washes. The pellet was resuspended in a small amount of electroporation buffer. The volume was adjusted to bring the final OD_600_ to 22. The cells were split up into 50 µL aliquots, dipped into liquid nitrogen and stored at − 80 °C until use.

#### *E. coli* BL21

10 mL LB-Lennox medium (10 g/L tryptone, 5 g/L yeast extract, 5 g/L NaCl, pH 7) was inoculated with BL21(DE3) cells and incubated at 37 °C and 200 rpm overnight. 150 mL LB-Lennox medium was inoculated with the ONC to an OD_600_ of 0.05 and incubated in a shaking flask at 37 °C and 180 rpm to a final OD_600_ of 0.5–0.7 (approx. 2.5 h). The culture was then centrifuged at 3900×*g* for 20 min at 4 °C. The pellet was washed with electroporation buffer (10% glycerol) and centrifuged again (3900×*g*, 15 min, 4 °C). The wash was repeated three times to a total of four washes. The pellet was resuspended in a small amount of electroporation buffer. The volume was adjusted to bring the final OD_600_ to 16. The cells were split up into 50 µL aliquots, dipped into liquid nitrogen and stored at − 80 °C until use.

### Site directed mutagenesis

Primers were designed by NEB online tool (New England Biolabs 2021a) for introducing amber stop codons (TAG) to EYFP position Y151 and MlaC position K100 (Supporting Information, Table S1). For Site directed mutagenesis the protocol for PCR using Q5® High-Fidelity DNA Polymerase provided by NEB was used (New England Biolabs 2021b).

### Electroporation

Agar plates containing kanamycin (KAN) and chloramphenicol (CHA) were prepared for *E. coli* (20 g/L LB-Lennox, 15 g/L agar, 50 µg/mL KAN, 25 µg/mL CHA) and *V.* *natriegens* (20 g/L LB-Lennox, 5 g/L NaCl, 15 g/L agar, 100 µg/mL KAN, 12.5 µg/mL CHA).

An aliquot of electrocompetent cells was kept on ice. Plasmid DNA was dialysed against ultrapure water for 1 h (MF™ membrane filters 0.025 µm, Millipore). 1–3 µL of plasmid DNA (for pET-1a and pEVOL, respectively) was added to the cells. The mix was incubated on ice for 1 min. The cell-DNA mix was transferred to a chilled 1 mm electroporation cuvette (Biozym). Vmax was electroporated with a 900 V pulse (Eppendorf Eporator V1.01). BL21 was electroporated with a 2500 V pulse. Cells were immediately recovered for 1.5 h. Vmax was recovered in 650 µL Enhanced 2xYT medium at 30 °C, while BL21 is recovered in 650 µL SOC medium (2% tryptone, 0.5% yeast extract, 10 mM NaCl, 2.5 mM KCl, 10 mM Mg_2_Cl, 10 mM MgSO_4_, 20 mM glucose) at 37 °C. Aliquots of the recovery medium were plated out on warm agar plates containing KAN for selection of clones containing the pET-1a plasmid and CHA for selection of clones containing the pEVOL plasmid. The plates were incubated over night at 30 °C (Vmax) or 37 °C (BL21) for colonies to appear. The transformation efficiencies for Vmax were in the range of 5–9 × 10^3^ cfu/µg.

### Plasmid purification and sequencing

The GeneJET Plasmid Miniprep Kit (Thermo Fisher, USA) was used and plasmid purification was done as described in the protocol. Purified plasmid DNA was sequenced by LGC genomics (Germany).

### Optimization of buffer components

#### *V. natriegens* Vmax

The concentration of buffer components was optimized for EYFP and MlaC expression according to the protein expression procedure of Becker et al. (2019). EYFP and MlaC were expressed in 25 mL M9 minimal medium (Supporting Information, Table S3 + S4) with NaCl and buffer components 1x (50 mM Na_2_HPO_4_, 25 mM KH_2_PO_4_, 10 mM NaCl, pH 8.0), 3x, 6x or 8x concentrated. Protein bands were compared on 12% Bis–Tris SDS-PAGE gels and M9 medium with best results was selected for all following experiments.

### Protein expression in 96-well-plates

#### *E. coli* BL21

10 mL 1xM9 minimal medium (+ 50 µg/mL KAN, + 25 µg/mL CHA) was inoculated with ONCs to an OD_600_ of 0.05. For each WT/MT three cultures from individual ONCs were prepared. Cells were incubated at 37 °C and 180 rpm and grown to a final OD_600_ of approx. 1.4. Then cell suspensions were transferred to a 96-well-plate (Perkin Elmer, black, clear bottom). Each culture was tested in quartets (4 wells) (Supporting Information, Figure S2). 0.02% arabinose, 1 mM IPTG and 2 mM AzF was added (1 M NaOH was added to WT and negative control instead of AzF) and fluorescence measurement was started immediately.

#### *V. natriegens* Vmax

10 mL 3xM9 minimal medium (+ 200 µg/mL KAN, + 25 µg/mL CHA) was inoculated with over ONCs to an OD_600_ of 0.05. For each WT/MT three cultures from individual ONCs were prepared. Cell suspensions were transferred to a 96-well-plate. Each culture was tested in quartets (4 wells). Cells were incubated at 30 °C while shaking. 0.02% arabinose was added 30 min after inoculation. 2 mM AzF was added 90 min after inoculation (1 M NaOH was added to WT and negative control instead of AzF) and 1 mM IPTG was added 120 min after inoculation. Fluorescence measurement was started immediately.

### Fluorescence measurement

1xM9 (BL21) or 3xM9 (Vmax) medium was used for blanks. Blank I was treated like WT/negative control. Blank II was treated like the experiments. All wells contained a final volume of 220 µL and plates were covered with sterile, gas permeable sealing (AeraSeal™, Excel Scientific, USA). 96-well plates were incubated at 25 °C with orbital shaking over night and fluorescence was measured every 20 min for approx. 21 h with excitation filter 485/20 nm and emission filter 535/25 nm (Beckman Coulter DTX 880). Fluorescence images were recorded with UV Gel Studio (Analytic Jena). Final values were normalized by OD_600_. Outliers were eliminated with a modified Thompson Tau Test. A Shapiro–Wilk test was used to test the data sets for normal distribution. The statistical significance was determined by an ordinary one-way ANOVA.

### Protein expression in shaking flasks

#### *E. coli* BL21

ONCs were prepared by inoculating 10 mL LB-Lennox (+ 50 µg/mL KAN, + 25 µg/mL CHA) with BL21(DE3) cells. ONCs were incubated at 37 °C and 200 rpm overnight. 25, 250 or 500 mL 1xM9 minimal medium (Supporting Information, Table S3 + S4) (+ 50 µg/mL KAN, + 25 µg/mL CHA) were inoculated with ONCs to an OD_600_ of 0.05 and incubated in shaking flasks at 37 °C and 180 rpm. Cells were grown to late growth phase (OD_600_ ~ 1.4) before 0.02% arabinose, 1 mM IPTG and 2 mM AzF was added (dissolved in 1 M NaOH) (not added to WT and negative control). Proteins were expressed at 25 °C and 180 rpm over night.

#### *V. natriegens* Vmax

ONCs were prepared by inoculating 10 mL enhanced 2xYT medium (+ 200 µg/mL KAN, + 25 µg/mL CHA) with Vmax cells. ONCs were incubated at 30 °C and 200 rpm over night. 25, 250 or 500 mL 3xM9 minimal medium (Supporting Information, Table S3 + S4) (+ 200 µg/mL KAN, + 25 µg/mL CHA) was inoculated with ONCs to an OD_600_ of 0.05 and incubated in shaking flasks at 30 °C and 180 rpm. 0.02% arabinose was added 30 min after inoculation. 2 mM AzF was added 90 min after inoculation (not added to WT and negative control) and 1 mM IPTG was added 120 min after inoculation. Proteins were expressed at 25 °C and 120 rpm over night.

### Protein purification (250/500 mL cultures)

Cells were pelleted at 3900×*g*, resuspended in 20 mL buffer W1 (50 mM NaPi buffer pH 8.0, 10 mM imidazole, 300 mM NaCl) with 40–100 µL protease inhibitor (Mix HP, Serva) and lysed by sonication (45% amplitude, 2 s on, 2 s off) for 20 min on ice. Cell lysates were centrifuged for 1 h at 20,000×*g* and supernatant was loaded onto a pre-equilibrated Ni^2+^-NTA gravity column. The column was washed with buffer W1, W3 (50 mM NaPi buffer pH 8.0, 10 mM imidazole, 1 M NaCl) and W4 (50 mM NaPi buffer pH 8.0, 20 mM imidazole, 300 mM NaCl) sequentially. The elution of His-bound protein was done with 10 mL elution buffer (50 mM NaPi buffer pH 8.0, 330 mM imidazole, 300 mM NaCl). 1–2 mg TEV protease and 5 mM 2-mercaptoethanol were added to the protein sample. Cleavage with TEV protease was performed over night at 4 °C while dialysed against buffer (20 mM Tris–HCl pH 8.0, 0.5 mM EDTA, 5 mM 2-mercaptoethanol, 150 mM NaCl) in a 3.5 kDa dialysis tube. The sample was loaded onto a pre-equilibrated Ni^2+^-NTA gravity column again. Washing and elution steps were done as described before. The flow-through was collected and loaded onto a pre-equilibrated size exclusion column (HiLoad 26/600 Superdex 75, GE Healthcare) and the protein was eluted with SEC buffer (50 mM NaPi pH 8.0, 300 mM NaCl) into 4 mL fractions. Fractions containing the pure protein were pooled and concentrated with 15 mL centrifugal filters (Amicon Ultra, MWCO 3000, Millipore).

### Sample preparation for SDS-PAGE (25 mL cultures)

Cells were pelleted at 3900×*g*, washed once with 1xPBS, resuspended in 3 mL lysis buffer (50 mM Tris–HCl pH 8.0, 100 mM NaCl) supplemented with 10 µL protease inhibitor and vortexed. Cells were lysed by sonication (40% amplitude, 2 s on, 3 s off) for 5 min on ice. 2 mL of cell lysate was centrifuged for 60 min at 4 °C (16,600×*g*). Supernatant was diluted 1:5 with lysis buffer. Pellets were resuspended in 2 mL 8 M urea and spun down for 5 min (16,600×*g*).

### SDS-PAGE

Samples were prepared with 4xLaemmli buffer, heated (100 °C, 5 min) for denaturation and applied onto a 12% Bis–Tris SDS-PAGE gel (NuPAGE™, Thermo Fisher). Gel electrophoresis was run at 150 V for 60 min. Gels were stained with a standard Coomassie staining protocol.

### Total and specific protein concentration determination

The total protein concentration was determined with the Pierce™ BCA Protein Assay Kit (Thermo Fisher). The test-tube procedure was used as described. The samples were measured on a NanoDrop 2000 (Thermo Fisher) at 562 nm. The specific protein concentration was determined by measuring 2 µL of protein sample at 280 nm with the specific extinction coefficient of EYFP (21,890 M^−1^ cm^−1^) and MlaC (31,400 M^−1^ cm^−1^). A Shapiro–Wilk test was used to test the data sets for normal distribution. The statistical significance was determined by an ordinary one-way ANOVA.

### Coupling of ^19^F-tags

For the coupling of ^19^F-tags the protocol for copper-catalyzed azide-alkyne cycloaddition of Presolski (2018) was used. 1 mM solutions of 2-(4-fluorphenyl)-3-butin-2-ol and 4-ethinyl-α,α,α-trifluortoluol were prepared in DMSO. After the coupling reaction proteins were dialysed against NMR buffer (50 mM NaPi pH 6.5, 50 mM NaCl) over night.

### NMR measurements

2D ^1^H-^15^N HSQC spectra of ~ 100 µM protein in NMR buffer were recorded in 3 mm NMR tubes at 25 °C with a 700 MHz Bruker Avance III NMR spectrometer equipped with a cryogenically cooled 5 mm TCI probe using z-axis gradients. Spectra were processed with NMRPipe (NMRDraw Version 5.6 Rev 2011.069.19.56).

^19^F-NMR spectra were recorded with a 500 MHz Bruker Avance III NMR spectrometer, equipped with a 5 mm ^19^F-H probe. ^19^F-spectra of ~ 115 µM tag-coupled protein and pure tags in 90% NMR buffer/10% D_2_O were recorded with the same settings and processed with MestreNova12.

### Protein digests

10 µg protein was reduced with 10 mM TCEP and alkylated with 40 mM chloroacetamide for 10 min (550 rpm, 95 °C). Digest was performed with 0.4 µg of modified trypsin (Promega) or 0.4 µg chymotrypsin (Promega) over night (550 rpm, 37 °C). 2 µg digest mix in 100 µL dH_2_O with 1% trifluoroacetic acid was loaded onto 2-layer 200 µL SDB-RPS Stage Tips (Empore SPE Disks, Sigma-Aldrich). The samples were passed through by centrifugation (5 min, 1500×*g*), washed with 0.2% trifluoroacetic acid, eluted with 5% NH_4_OH/80% acetonitrile (ACN) and dried using a vacuum concentrator. 100 ng sample was dissolved in dH_2_O with 2% ACN and 0.1% formic acid (FA) and subjected to LC–MS/MS analysis. Protein digests were separated by Nano-HPLC (Dionex Ultimate 3000, Thermo Fisher Scientific) equipped with an Aurora nanocolumn (C18, 1.6 µm, 250 × 0.075 mm, Ionoptics) at a flow rate of 300 nL/min at 50 °C using a gradient elution (A: 0.1% FA in dH_2_O, B: 0.1% FA in ACN, gradient 2–95% B). Samples were measured on a maXis II ETD mass spectrometer (Bruker Daltonics) equipped with a captive source in positive mode employing following settings: mass range: 200–2000 m/z, 2 Hz, capillary 1600 V, dry gas flow 3 L/min at 150 °C, nanoBooster 0.2 bar, precursor acquisition control top 20 (collision induced dissociation). The LC–MS/MS data were analysed by searching a database containing all protein sequences and all common contaminants with Mascot 2.3 (MatrixScience) (Supporting Information, Method Supplementary p10).

### Intact mass measurement

The protein solutions were desalted using a 0.5 mL centrifugal filter (Amicon Ultra, MWCO 3000, Millipore). Samples were concentrated to a final concentration of 1 pmol/µL in dH_2_O containing 50% ACN and 0.1% FA. Samples were measured on a maXis II ETD mass spectrometer equipped with an ESI source in positive mode with the following settings: Mass range 250–3000 m/z, 1 Hz, source voltage 4.5 kV, dry gas flow 4 L/min at 180 °C. Deconvolution of protein mass spectra was done by the MaxEnt algorithm. The following settings were used: Charge carrier H^+^, m/z range 800–2000, min. instrument resolving power 50,000. For peak detection SNAP algorithm with following parameters was used: Quality factor threshold 0.9, S/N threshold 2 and maximum charge state of 12. For the following samples a modified method was used: Vmax EYFP-WT pAzFRS.2.t1, Vmax EYFP-Y151AzF pAzFRS.2.t1 (Supporting Information, Method Supplementary p10).

## Supplementary Information

Below is the link to the electronic supplementary material.

Supplementary file1 (DOCX 1423 KB)

## Data Availability

Supporting Information: Protein and primer sequences, synthetase mutations, M9 medium components, SDS-PAGE gel for M9 medium optimization, experimental details of 96-well-plate expressions, total and specific protein amounts, protein expression details, mass spectroscopy data, NMR spectra of ^19^F-tags, method supplementary.

## Abbreviations

AzF: p-azido-L-phenylalanine
HSQC: Heteronuclear Single Quantum Coherence
IPTG: Isopropyl-β-D-thiogalactopyranosid
ncAA: Non-canonical amino acid
PrOF NMR: Protein-observed 19F NMR
SE: Suppression efficiency
