# Supplementary material for: Overexpression of recombinant proteins containing non-canonical amino acids in Vibrio natriegens: p-azido-L-phenylalanine as coupling site for 19F-tags
Source: Amino Acids. 2022 Apr 13;54(7):1041–53. doi: 10.1007/s00726-022-03148-2 (PMC9217835; doi:10.1007/s00726-022-03148-2)
Supplement: Supplementary file 1 — Supplementary file1 (DOCX 1423 KB) [file 726_2022_3148_MOESM1_ESM.docx]

Overexpression of Recombinant Proteins Containing Non-Canonical Amino Acids in *Vibrio natriegens*: *p*-Azido-L-phenylalanine as Coupling Site for ^19^F-tags

Karina A. Stadler^1^, Walter Becker^1,+^, Barbara Darnhofer^2,3^, Ruth Birner-Gruenberger^2,3,4^, Klaus Zangger^1,^*

^1^ Institute of Chemistry, University of Graz, Heinrichstrasse 28, 8010 Graz, Austria

^2^ Diagnostic and Research Institute of Pathology, Medical University of Graz, Stiftingtalstrasse 6, 8010 Graz, Austria

^3^ Omics Center Graz, BioTechMed-Graz, Stiftingtalstrasse 24, 8010 Graz, Austria

^4^ Institute of Chemical Technologies and Analytics, Faculty of Technical Chemistry, Technische Universität Wien, Getreidemarkt 9/164, 1060 Vienna, Austria

^+^ Karolinska Institutet, Department of Medical Biochemistry & Biophysics, Solnavägen 9, 17177 Stockholm, Sweden

* Corresponding author: klaus.zangger@uni-graz.at

Supporting Information

**Protein sequences**

The proteins EYFP and MlaC were expressed in 6xHis-Z2/NusA-TEVsite-protein constructs. The protein sequences after TEV cleavage are shown in Table S1. Amber stop mutations were inserted at specific sites with site directed mutagenesis, using the primers shown below.

Table S1: Protein sequences, incorporation sites and primer for site directed mutagenesis

|  | **EYFP**  **UniProtKB: P42212** | **MlaC**  **UniProtKB: P45028[29 - 214]** |
| --- | --- | --- |
| Construct | 6xHis-Z2-TEVsite-EYFP (37.4 kDa) | 6xHis-NusA-TEVsite-MlaC (78.8 kDa) |
| Wildtype sequence  (after TEV cleavage) | GAMGKVSKGEELFTGVVPILVELDGDVNGHKFSVSGEGEGDATYGKLTLKFICTTGKLPVPWPTLVTTFGYGLQCFARYPDHMKQHDFFKSAMPEGYVQERTIFFKDDGNYKTRAEVKFEGDTLVNRIELKGIDFKEDGNILGHKLEYNYNSHNVYIMADKQKNGIKVNFKIRHNIEDGSVQLADHYQQNTPIGDGPVLLPDNHYLSTQSALSKDPNEKRDHMVLLEFVTAAGITLGMDELYK | GAMETSPYVLMQQAADKLFSDIQANQSKIKQDPNYLRTIVRNDLLPYVNLEYAGSKVLGSYYKSTSAEQREKFFKTFGELIEQKYAQALTNYSNQKIQIESEKELGDNNFINIRVNIIQANGVAPILLYFKWRKGNKSGEWKVYDMVGAGVSMLEDTIKNWVGILNKQGIDTLITKMQQSASQPIIFNQ |
| Incorporation site | Y151* | K100* |
| Mutant sequence  (after TEV cleavage) | GAMGKVSKGEELFTGVVPILVELDGDVNGHKFSVSGEGEGDATYGKLTLKFICTTGKLPVPWPTLVTTFGYGLQCFARYPDHMKQHDFFKSAMPEGYVQERTIFFKDDGNYKTRAEVKFEGDTLVNRIELKGIDFKEDGNILGHKLEYNYNSHNV*IMADKQKNGIKVNFKIRHNIEDGSVQLADHYQQNTPIGDGPVLLPDNHYLSTQSALSKDPNEKRDHMVLLEFVTAAGITLGMDELYK | GAMETSPYVLMQQAADKLFSDIQANQSKIKQDPNYLRTIVRNDLLPYVNLEYAGSKVLGSYYKSTSAEQREKFFKTFGELIEQKYAQALTNYSNQKIQIESE*ELGDNNFINIRVNIIQANGVAPILLYFKWRKGNKSGEWKVYDMVGAGVSMLEDTIKNWVGILNKQGIDTLITKMQQSASQPIIFNQ |
| Forward Primer | CCACAACGTCtagATCATGGCCG | TGAATCAGAAtagGAATTAGGCGATAAC |
| Reverse Primer | CTGTTGTAGTTGTACTCCAGC | ATTTGAATTTTTTGATTAGAATAATTTG |

**Synthetase mutations**

The two synthetase variants used in this study differ from the original *M. jannaschii* AzF-tRNA synthetase by the following mutations:

Table S2: Mutations of synthetase variants in pEVOL plasmids

| **Plasmids** | **pEVOL-pAzF** | **pEVOL-pAzFRS.2.t1** |
| --- | --- | --- |
| Synthetase mutations | Y32T, E107N, D158P, I159L, L162Q, D286R | E107T, F108Y, Q109M, R257G |

**M9 minimal medium**

Table S3 shows components and concentrations of M9 minimal medium with 1x, 3x, 6x and 8x concentrated NaCl and buffer components. Table S4 shows the components and concentrations of microsalts stock (1000x) and vitamins stock (1000x).

Table S3: Components and concentrations of M9 minimal medium

| **M9 buffer components** | **1x** | **3x** | **6x** | **8x** |
| --- | --- | --- | --- | --- |
| Na_2_HPO_4_  KH_2_PO_4_  NaCl  pH | 50 mM  25 mM  10 mM  8.0 | 150 mM  75 mM  30 mM  8.0 | 300 mM  150 mM  60 mM  8.0 | 400 mM  200 mM  80 mM  8.0 |
| **General components** |  | | | |
| CaCl_2_  MgSO_4_  Microsalts (from stock)  Vitamins (from stock)  ^14^N/^15^NH_4_Cl  ^12^C/^13^C-Glucose | 0.2 mM  5 mM  0.1% (v/v)  0.1% (v/v)  0.15% (w/v)  1.00% (w/v) | | | |

| **Microsalts stock 1000x** | **Concentration** |
| --- | --- |
| ZnCl_2_  MgCl_2_  CaCl2  H_3_BO_3_  FeCl_3_  CuCl_2_  CoCl_2_  (NH_4_)_6_Mo_7_O_24_ | 1500 mM  1000 mM  150 mM  50 mM  20 mM  800 µM  150 µM  15 µM |
| **Vitamins stock 1000x** | **Concentration** |
| Biotin  Thiamine | 1 mg/mL  1 mg/mL |

Table S4: Components and concentrations of microsalts stock (1000x) and vitamins stock (1000x)

**Optimization of NaCl and buffer components in M9 minimal medium**

Proteins EYFP and MlaC were expressed in M9 minimal medium containing 1x, 3x, 6x and 8x concentrated NaCl and buffer components. 1:5 diluted cell lysates were applied to 12% Bis-Tris SDS-PAGE gels. The uncleaved protein construct of EYFP (see Table S1) appears between 34 and 43 kDa in Figure S1A. The uncleaved protein construct of MlaC (see Table S1) appears between 72 and 95 kDa in Figure S1B. The strongest expression was observed in 3xM9 medium for both proteins.


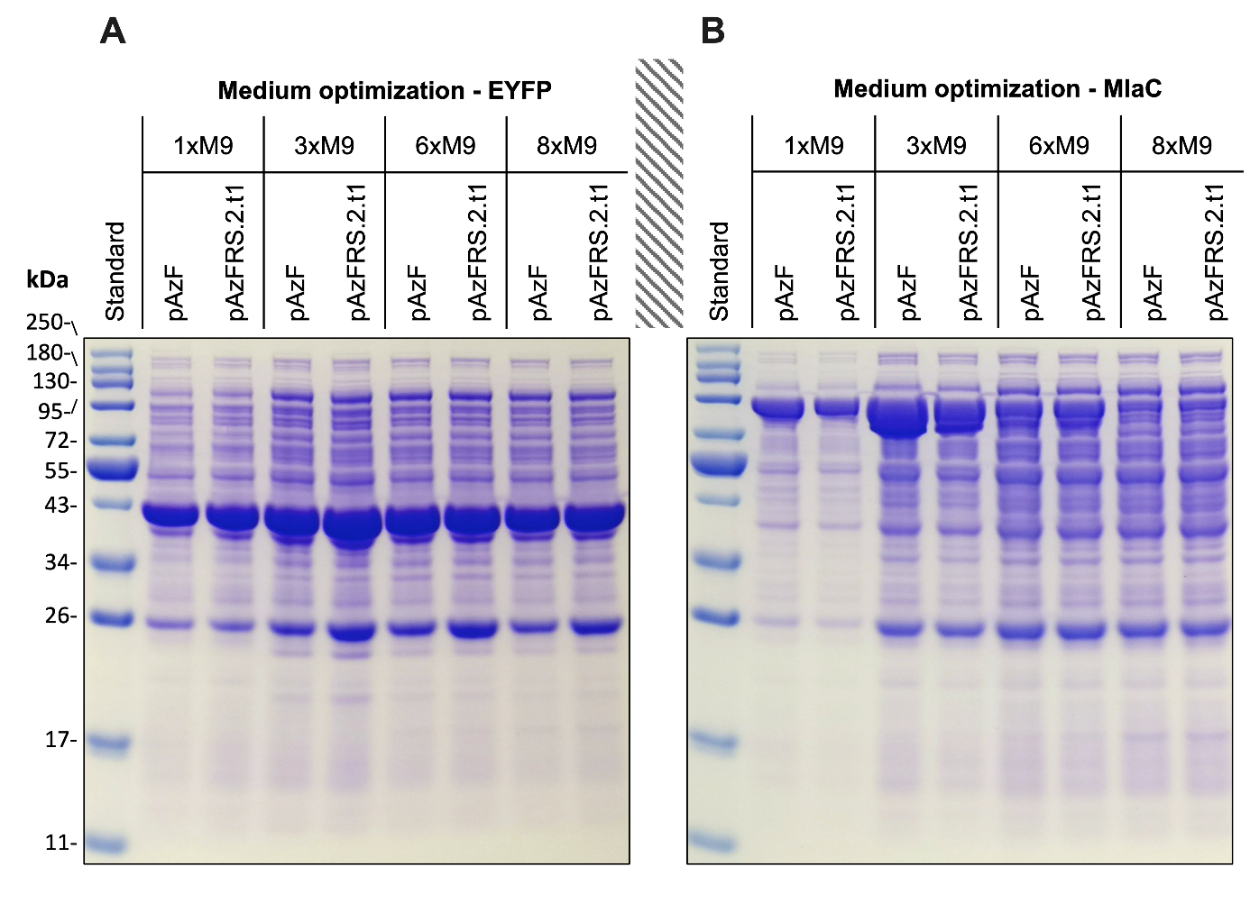


Figure S1: Optimization of NaCl and buffer components. (A) SDS-PAGE gel showing the strongest expression for EYFP in 3xM9 medium. (B) SDS-PAGE gel showing the strongest expression of MlaC in 3xM9 medium.

**96-well-plate expressions**

Figure S2 shows the pipetting scheme for 96-well-plate expressions. Arabinose, AzF/NaOH and IPTG were added accordingly to reach a final volume of 220 µL per well.


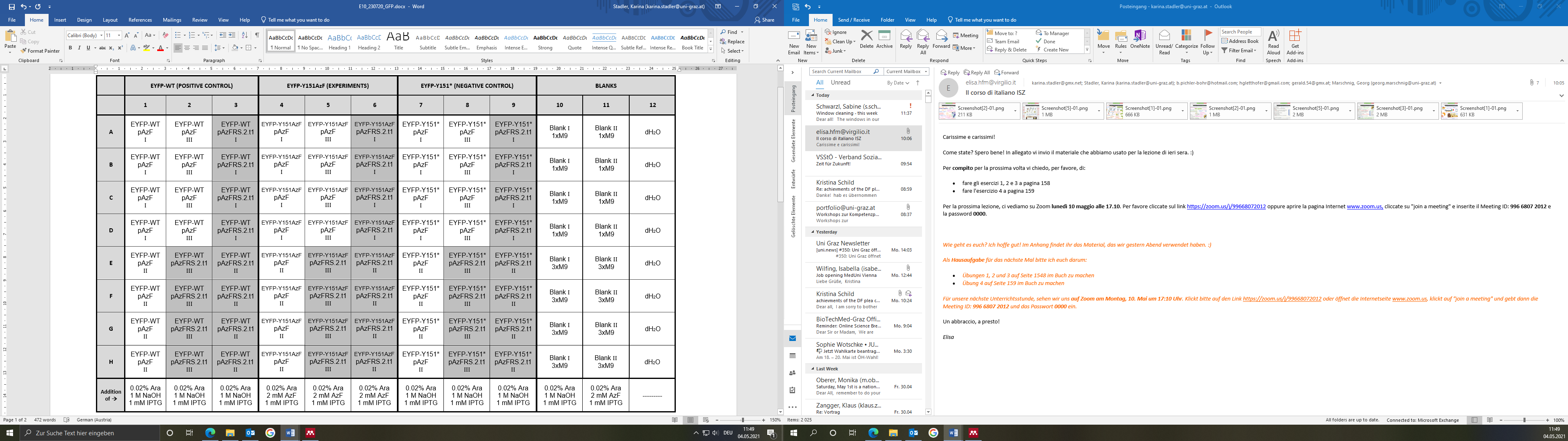


Figure S2: Pipetting scheme for 96-well-plate expressions

**Results from 96-well-plate expressions**

Relative fluorescence units were measured, normalized by the OD_600_ values in each well. Table S5 shows the values divided by 1000. The mean, standard deviation and suppression efficiencies (SE) were calculated from *n* = 3 independently grown cultures (*p* < 0.0001).

Table S5: Results from 96-well-plate expressions

|  | | | **Mean**  **[RFU/OD_600_ x1000]** | **STD** | **SE [%]** | **STD [%]** |
| --- | --- | --- | --- | --- | --- | --- |
| ***V. natriegens* Vmax Express** | **pAzF** | EYFP-WT | 40950 | 1260 | 100.0 | 3.1 |
|  |  | EYFP-Y151AzF | 6342 | 327 | 15.5 | 0.8 |
|  |  | EYFP-Y151* | 141 | 2 | 0.3 | < 0.0 |
|  | **pAzFRS.2.t1** | EYFP-WT | 32860 | 1190 | 100.0 | 3.6 |
|  |  | EYFP-Y151AzF | 11599 | 267 | 35.3 | 0.8 |
|  |  | EYFP-Y151* | 576 | 109 | 1.8 | 0.3 |
| ***E. coli***  **BL21(DE3)** | **pAzF** | EYFP-WT | 68701 | 126 | 100.0 | 0.2 |
|  |  | EYFP-Y151AzF | 42152 | 1865 | 61.4 | 2.7 |
|  |  | EYFP-Y151* | 340 | 15 | 0.5 | < 0.0 |
|  | **pAzFRS.2.t1** | EYFP-WT | 66055 | 970 | 100.0 | 1.5 |
|  |  | EYFP-Y151AzF | 5430 | 176 | 8.2 | 0.3 |
|  |  | EYFP-Y151* | 316 | 15 | 0.5 | < 0.0 |

**Total and specific protein amount of EYFP**

The total protein amount was determined by the Pierce™ BCA Protein Assay Kit (Thermo Fisher) from pure protein samples. Absorbance values were converted to protein concentration values by the linear equation obtained from the standard curve. Values were corrected by dilution factor and volume. The mean, STD and SE were calculated from *n* = 3 measuring repeats (*p* < 0.0001).

Table S6: Total protein amount and suppression efficiencies of EYFP

| *Linear equation: y=1.0568x+0.1282 (R²=0.9984) | | **Absorbance** | | | | | **Conc. [mg/mL]*** | | | | **Dilution factor** | | | | **Volume** |
| --- | --- | --- | --- | --- | --- | --- | --- | --- | --- | --- | --- | --- | --- | --- | --- |
|  |  | 1 | 2 | | 3 | | 1 | 2 | 3 | | 1 | 2 | | 3 | **[mL]** |
| **Vmax Express** | **EYFP-WT** | 0.80 | 0.82 | | 0.81 | | 0.64 | 0.66 | 0.65 | | 1.28 | 1.31 | | 1.29 | 48 |
|  | **EYFP-Y151AzF** | 0.29 | 0.29 | | 0.28 | | 0.15 | 0.15 | 0.15 | | 0.31 | 0.31 | | 0.29 | 44 |
| **BL21(DE3)** | **EYFP-WT** | 0.61 | 0.62 | | 0.61 | | 0.46 | 0.47 | 0.46 | | 0.91 | 0.93 | | 0.91 | 44 |
|  | **EYFP-Y151AzF** | 0.42 | 0.43 | | 0.42 | | 0.28 | 0.29 | 0.28 | | 0.55 | 0.57 | | 0.55 | 40 |
|  | | **Total protein amount [mg]** | | | | | | | | | | |  | | |
|  | | 1 | | 2 | | 3 | | **Mean [mg]** | | **STD [mg]** | | | **SE [%]** | | **STD [%]** |
| **Vmax Express** | **EYFP-WT** | 61.39 | | 62.93 | | 61.93 | | 62.09 | | 0.78 | | | 100 | | 0.0 |
|  | **EYFP-Y151AzF** | 13.56 | | 13.56 | | 12.97 | | 13.36 | | 0.34 | | | 55.8 | | 1.1 |
| **BL21(DE3)** | **EYFP-WT** | 43.77 | | 44.68 | | 43.77 | | 44.07 | | 0.52 | | | 100 | | 0.0 |
|  | **EYFP-Y151AzF** | 24.30 | | 25.13 | | 24.30 | | 24.58 | | 0.48 | | | 21.5 | | 0.5 |

The specific protein amount was determined photometrically at 280 nm. Absorbance was converted by the NanoDrop 2000 software with the molecular weight (27.24 kDa) and extinction coefficient (21890 M^-1^cm^-1^) of EYFP to protein concentration. Values were normalized by the volume. The mean, STD and SE were calculated from *n* = 3 measuring repeats (*p* < 0.0001).

Table S7: Specific protein amount and suppression efficiencies of EYFP

| *EYFP: 27.24 kDa, Extinction coefficient 21890 M^-1^cm^-1^ | | **Concentration [mg/mL]*** | | | **Vol** | **Specific protein amount [mg]** | | | | |  | |
| --- | --- | --- | --- | --- | --- | --- | --- | --- | --- | --- | --- | --- |
|  |  | 1 | 2 | 3 | **[mL]** | 1 | 2 | 3 | **Mean [mg]** | **STD [mg]** | **SE [%]** | **STD [%]** |
| **Vmax Express** | **EYFP-WT** | 1.47 | 1.49 | 1.48 | 48 | 70.56 | 71.52 | 71.04 | 71.0 | 0.5 | 100 | 0.0 |
|  | **EYFP-Y151AzF** | 0.31 | 0.33 | 0.34 | 44 | 13.64 | 14.52 | 14.96 | 14.4 | 0.7 | 59.8 | 1.4 |
| **BL21(DE3)** | **EYFP-WT** | 1.06 | 1.00 | 0.96 | 48 | 50.88 | 48.00 | 46.08 | 48.3 | 2.4 | 100 | 0.0 |
|  | **EYFP-Y151AzF** | 0.66 | 0.64 | 0.67 | 44 | 29.04 | 28.16 | 29.48 | 28.9 | 0.7 | 20.2 | 0.9 |

**Protein expression details**

During protein expression of EYFP the following data was collected:

Table S8: Protein expression details of EYFP

|  | **Protein** | **Glucose [g]** | **Volume [mL]** | **Wet cell weight [g]** | **OD_600_** | **pH** |
| --- | --- | --- | --- | --- | --- | --- |
| **Vmax Express** | EYFP-WT pAzFRS.2.t1 | 5 | 500 | 5.85 | 4.9 | 6.6 |
|  | EYFP-Y151AzF pAzFRS.2.t1 |  |  | 5.84 | 3.7 | 6.6 |
| **BL21(DE3)** | EYFP-WT pAzF |  |  | 3.49 | 3.8 | 6.7 |
|  | EYFP-Y151AzF pAzF |  |  | 3.85 | 2.7 | 6.7 |

**Mass spectroscopic measurements of EYFP**

Table S9 shows the results of mass spectroscopic measurements of protein digest fragments. The fragments containing the position 151 (K.LEYNYNSHNVYIMADK.Q) have a monoisotopic mass of 1972.9 Da. The fragments were found either with no modification, the AzF-modification and/or oxidations with corresponding monoisotopic masses.

Table S9: Mass spectroscopic measurement of protein digest fragments of EYFP

| **Strain** | **Protein** | **Sequence coverage [%]** | **RMS90 [ppm]** | **Modifications** | **Number of Compounds** | **Monoisotopic mass [Da]** | **Δ m/z [ppm]** | **RMS90 [ppm]** | **Scores** |
| --- | --- | --- | --- | --- | --- | --- | --- | --- | --- |
| ***V. natriegens* Vmax Express** | **EYFP-WT pAzFRS.2.t1** | 58.0 | 2.45 | No modification | 3 | 1972.9 | -2.8 | 5.94 | 102.1 |
|  |  |  |  | Oxidation | 2 | 1988.9 | -3.38 | 2.89 | 126.4 |
|  | **EYFP-Y151AzF pAzFRS.2.t1** | 42.0 | 2.81 | AzF | 10 | 1971.9 | -4.91 | 32782 | 106.7 |
|  |  |  |  | AzF, Oxidation | 7 | 1987.9 | -6.51 | 15.66 | 32.7 |
| ***E. coli* BL21(DE3)** | **EYFP-WT pAzF** | 43.2 | 0.89 | No modification | 19 | 1972.9 | 0.90 | 4.87 | 39.4 |
|  |  |  |  | Oxidation | 11 | 1988.9 | 1.93 | 8.10 | 38.5 |
|  | **EYFP-Y151AzF pAzF** | 63.4 | 0.90 | AzF | 3 | 1971.9 | 0.59 | 3.71 | 58.3 |
|  |  |  |  | AzF, Oxidation | 2 | 1987.9 | 1.61 | 11.57 | 19.1 |

Table S10 shows the results of intact mass measurements of EYFP. EYFP-WT results in a mass of 27205.66 ± 0.02 Da, while EYFP-Y151AzF results in a mass of 27230.72 ± 0.05 Da. The mass difference of 25.06 Da matches with the AzF-modification.

Table S10: Intact mass measurements of EYFP

| **Strain** | **Protein** | **Mass calculated [Da]** | **Mass measured [Da]** | **Intensity** | **Residues** |
| --- | --- | --- | --- | --- | --- |
| **Vmax Express** | **EYFP-WT** | 27205.61 | 27205.68 | 505755 | 84320 |
|  | **EYFP-Y151AzF** | 27230.62 | 27230.75 | 3991068 | 69831 |
| **BL21(DE3)** | **EYFP-WT** | 27205.61 | 27205.65 | 480732 | 82570 |
|  | **EYFP-Y151AzF** | 27230.62 | 27230.68 | 505755 | 84320 |

**Specific protein determination of MlaC**

The specific protein amount was determined photometrically at 280 nm. Absorbance was converted by the NanoDrop 2000 software with the molecular weight (21.53 kDa) and extinction coefficient (31400 M^-1^cm^-1^) of MlaC to protein concentration. Values were normalized by the volume. The mean, STD and SE were calculated from *n* = 3 measuring repeats (*p* < 0.0001).

Table S11: Specific protein concentrations and SE of MlaC

| *MlaC: 21.53 kDa, Extinction coefficient 31400  M^-1^cm^-1^ | **Concentration [mg/mL]*** | | | Vol | **Specific protein amount [mg]** | | | | |  |  |
| --- | --- | --- | --- | --- | --- | --- | --- | --- | --- | --- | --- |
|  | 1 | 2 | 3 | [mL] | 1 | 2 | 3 | **Mean [mg]** | **STD [mg]** | **SE [%]** | **STD [%]** |
| **YrbC-WT** | 0.45 | 0.46 | 0.45 | 32 | 14.40 | 14.72 | 14.27 | 14.46 | 0.23 | 100.0 | 0.0 |
| **YrbC-K100AzF pAzF** | 0.03 | 0.03 | 0.03 | 24 | 0.70 | 0.62 | 0.62 | 0.65 | 0.04 | 4.5 | 0.3 |
| **YrbC-K100AzF pAzFRS.2.t1** | 0.10 | 0.09 | 0.10 | 28 | 2.66 | 2.60 | 2.91 | 2.73 | 0.16 | 18.8 | 1.1 |

**Protein expression details**

During protein expression of MlaC the following data was collected:

Table S12: Protein expression details of MlaC

|  | **Protein** | **Glucose [g]** | **Volume [mL]** | **OD_600_ after expression** | **pH after expression** |
| --- | --- | --- | --- | --- | --- |
| **Vmax Express** | EYFP-WT pAzF | 2.5 | 250 | 8.2 | 6.8 |
|  | EYFP-Y151AzF pAzF |  |  | 4.3 | 6.7 |
|  | EYFP-Y151AzF pAzFRS.2.t1 |  |  | 4.9 | 6.7 |

**Mass spectroscopic measurements of MlaC**

Mass spectroscopic analyses of MlaC was done with tryptic and chymotryptic digest. For MlaC-WT no modifications could be found in tryptic and chymotryptic fragments. In MlaC-K100AzF of both variants AzF-modifications were found in chymotryptic fragments. The trypsin digest revealed both, modified and unmodified fragments.

Table S13: Tryptic and chymotryptic digest results from MlaC

| **Protein** | **Sequence**  **coverage [%]** | **RMS90 [ppm]** | **Type of digest** | **Fragment** | **Modification** | **Number of**  **Compounds** | **Monoisotopic**  **mass [Da]** | **Δ m/z**  **[ppm]** | **RMS90**  **[ppm]** | **Scores** |
| --- | --- | --- | --- | --- | --- | --- | --- | --- | --- | --- |
| **MlaC-WT** | 87.3 | 0.97 | Trypsin | K.IQIESEK.E | No modification | 97 | 845.4 | -1.84 | 4.70 | 34.9 |
|  |  |  |  | K.IQIESEKELGDNNFINIR.V |  | 1 | 2131.1 | -1.76 | 9.69 | 19.6 |
|  |  |  | Chymotrypsin | Y.SNQKIQIESEKEL.G |  | 12 | 1544.8 | -1.75 | 4.10 | 50.7 |
|  |  |  |  | Y.SNQKIQIESEKELGDNNF.I |  | 6 | 2092.0 | -1.98 | 4.16 | 78.9 |
| **MlaC-K100AzF pAzF** | 87.8 | 0.93 | Trypsin | K.IQIESEK.E | No modification | 28 | 845.4 | -1.39 | 2.32 | 29.2 |
|  |  |  |  | K.IQIESEKELGDNNFINIR.V | AzF | 3 | 2164.2 | 0.43 | 9.56 | 40.8 |
|  |  |  | Chymotrypsin | Y.SNQKIQIESEKELGDNNF.I | AzF | 13 | 2126.0 | -0.85 | 6.36 | 83.9 |
| **MlaC-K100AzF pAzFRS.2.t1** | 87.8 | 1.22 | Trypsin | K.IQIESEK.E | No modification | 11 | 845.4 | -1.79 | 13.61 | 31.5 |
|  |  |  |  | K.IQIESEKELGDNNFINIR.V | AzF | 1 | 2165.1 | 0.29 | 8.52 | 19.1 |
|  |  |  | Chymotrypsin | Y.SNQKIQIESEKEL.G | AzF | 1 | 1578.8 | -0.91 | 24.77 | 35.7 |
|  |  |  |  | Y.SNQKIQIESEKELGDNNF.I | AzF | 20 | 2126.0 | -0.61 | 11.73 | 104.6 |

**NMR spectra of ^19^F-tags**

Figure S3 shows the ^19^F-NMR spectrum of pure (90%) 2-(4-fluorphenyl)-3-butin-2-ol (tag A) in 90% NMR buffer/10% D_2_O. The chemical was purchased with a purity of 90%. The larger peak between -115 and -116 ppm is the signal of tag A. Additionally, the spectrum shows a signal of a by-product at -105 ppm.


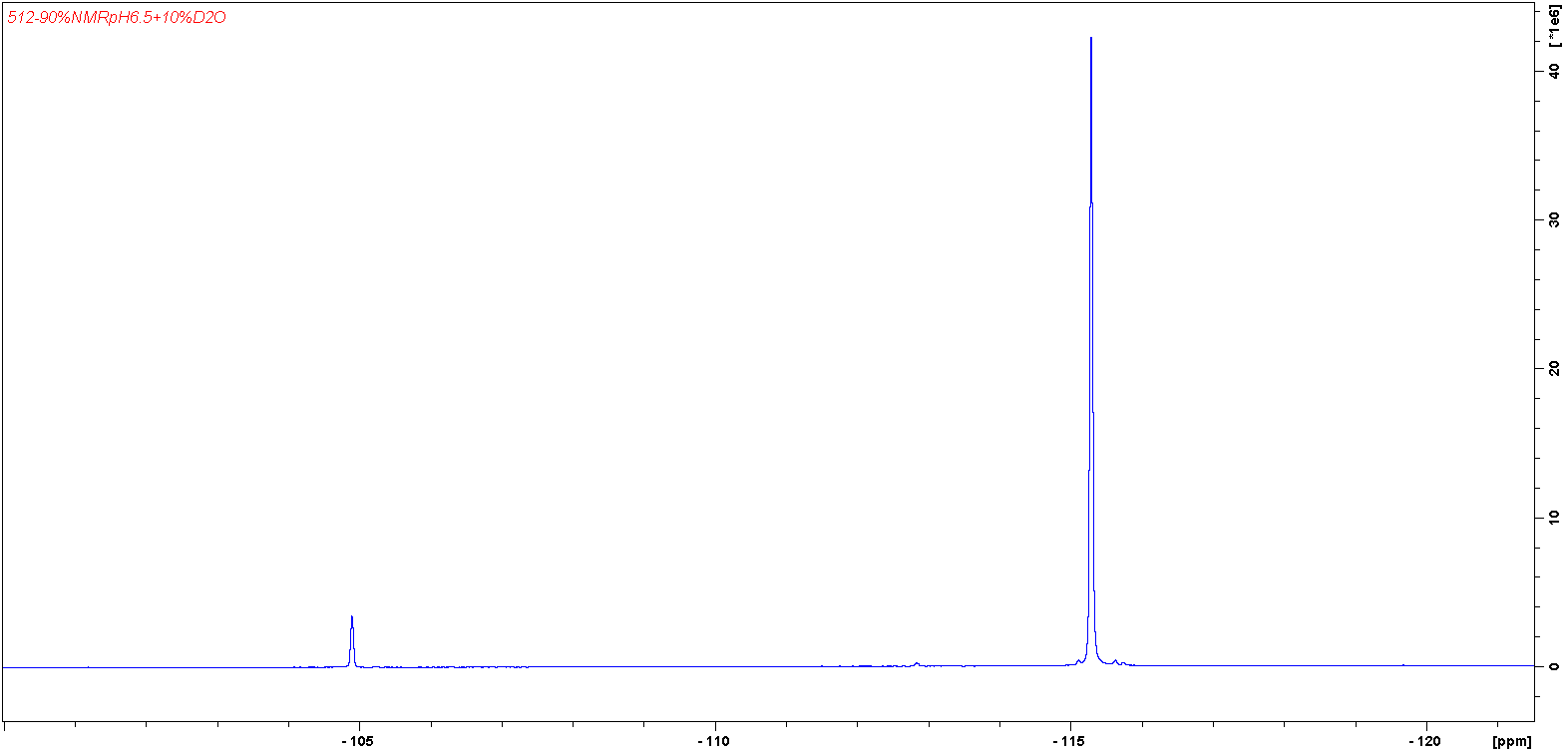


Figure S3: ^19^F-NMR spectrum of pure ^19^F-tag A

Figure S4 shows the ^19^F-NMR spectrum of tag A coupled to EYFP-Y151AzF in 90% NMR buffer/10% D_2_O. The spectrum shows a peak of the by-product (shifted to -119.5 ppm) and two broader peaks between -115 and -116 ppm, where one peak represents tag A coupled to EYFP-Y151AzF and one peak represents unbound tag A.


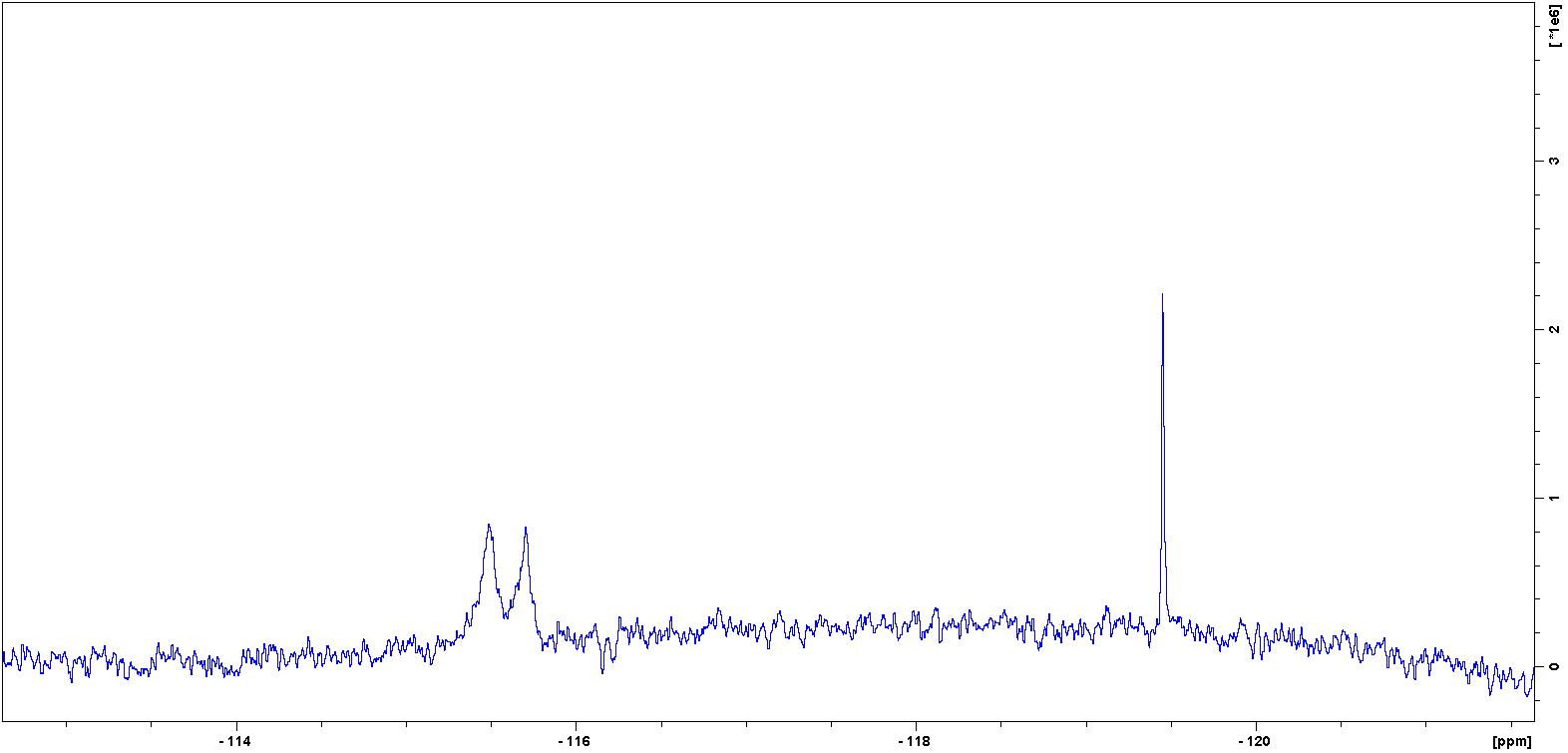


Figure S4: ^19^F-NMR spectrum of ^19^F-tag A coupled to EYFP-151AzF

Figure S5 shows the ^19^F-NMR spectrum of pure (97%) 4‑ethinyl-α,α,α-trifluortoluol (tag B) in 90% NMR buffer/10% D_2_O. The signal of tag B appears between -62 and -63 ppm.


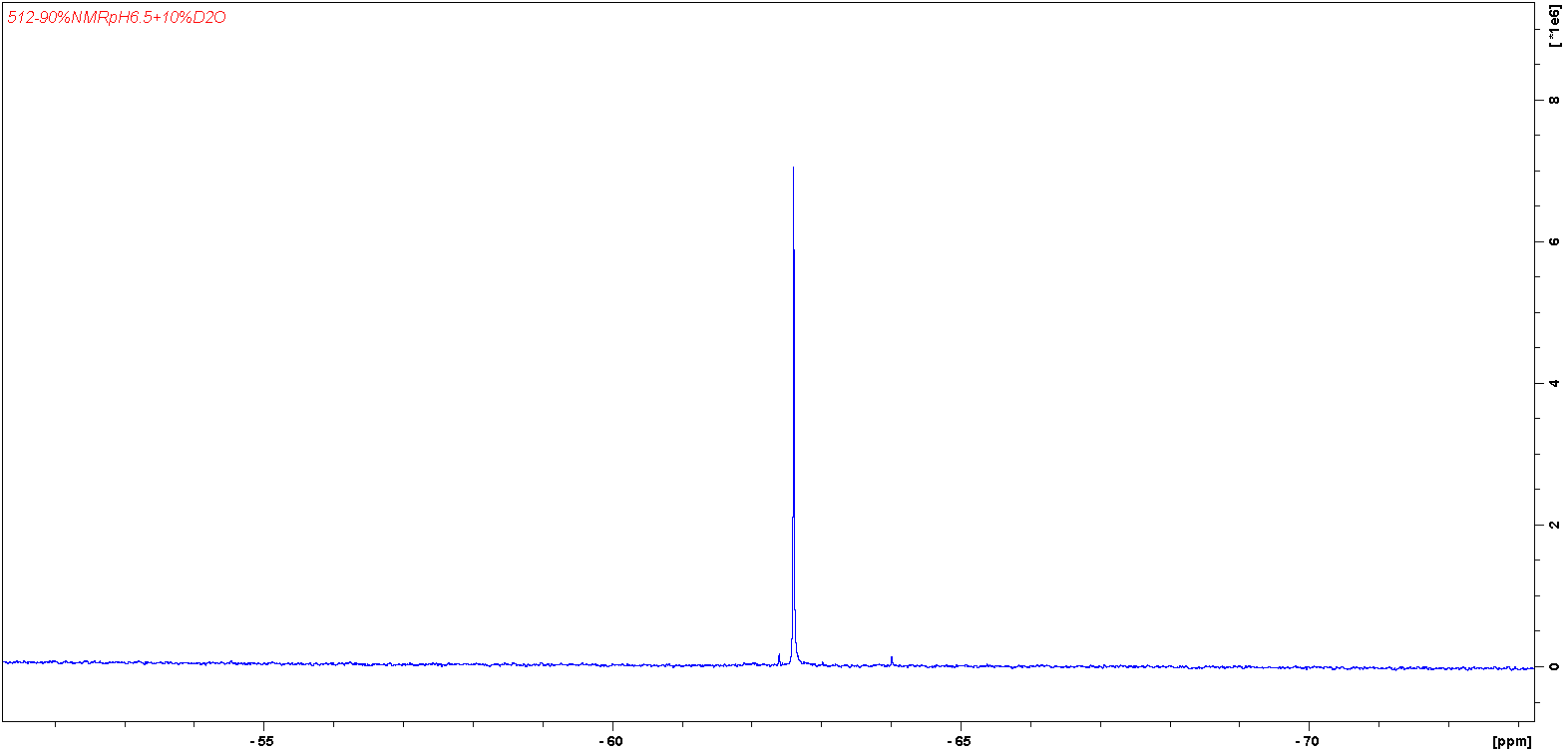


Figure S5: ^19^F-NMR spectrum of pure ^19^F-tag B

Figure S6 shows the ^19^F-NMR spectrum of tag B coupled to EYFP-Y151AzF in 90% NMR buffer/10% D_2_O. The spectrum shows two peaks between -62 and -63 ppm, where one peak represents tag B coupled to EYFP-Y151AzF and one peak represents unbound tag B.


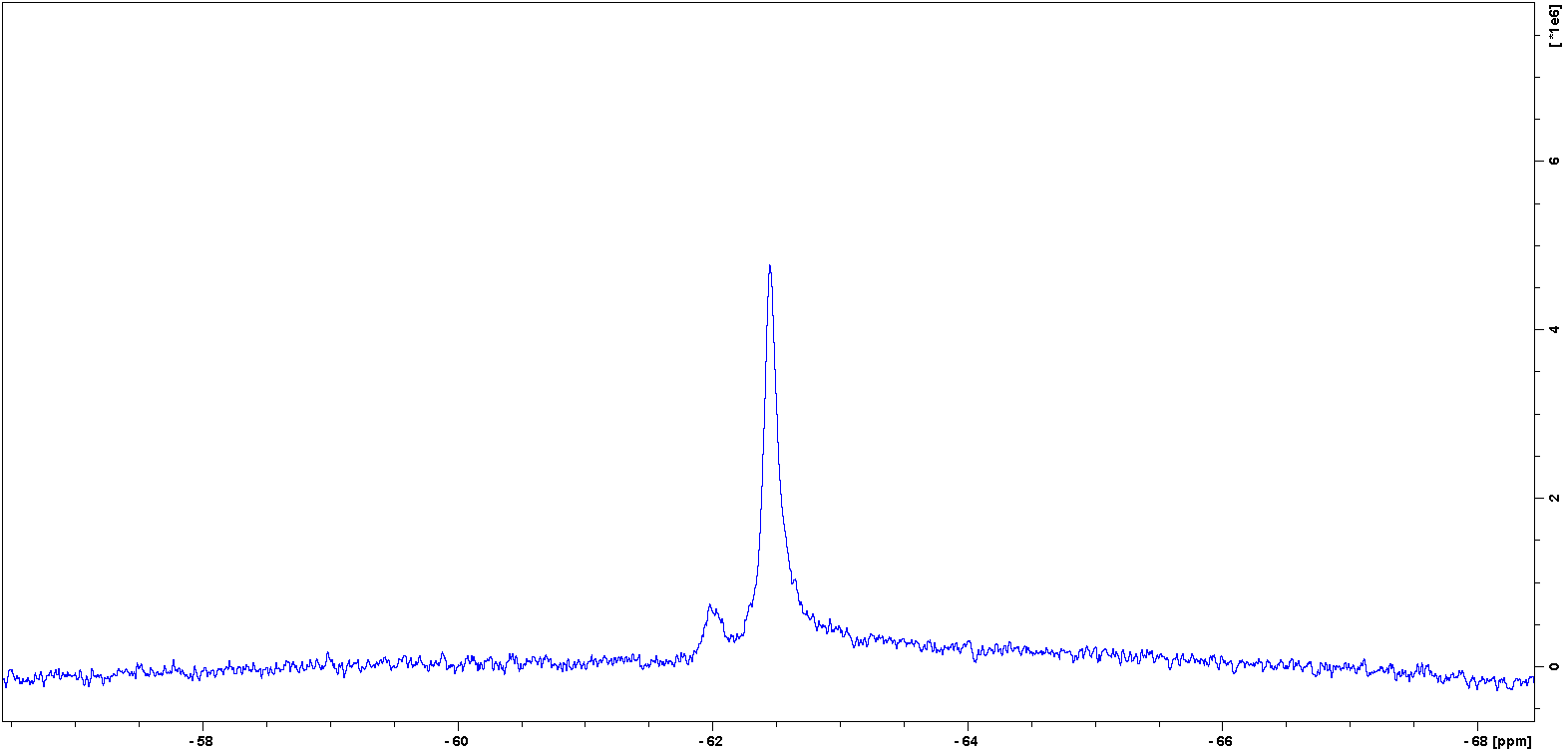


Figure S6: ^19^F-NMR spectrum of ^19^F-tag B coupled to EYFP-151AzF

**Intact mass measurements of EYFP coupled to ^19^F-tags**

Table S14 shows the results of intact mass measurements of EYFP coupled to ^19^F-tags. EYFP-AzF results in a mass of 27230.69 ± 0.03 Da, EYFP-Y151AzF-tag A results in a mass of 27394.73 Da and EYFP-Y151AzF-tag B results in a mass of 27400.67 Da. 2-(4-Fluorphenyl)-3-butin-2-ol (tag A) has a molar mass of 164.18 Da. The mass difference of EYFP-Y151AzF-tag A to EYFP-Y151AzF is 164.04 Da and confirms the coupling of tag A (0.14 Da deviation to expected mass). 4‑Ethinyl-α,α,α-trifluortoluol (tag B) has a molar mass of 170.13 Da. The mass difference of EYFP-Y151AzF-tag B to EYFP-Y151AzF is 169.98 Da and confirms the coupling of tag B (0.13 Da deviation to expected mass).

Table S14: Intact mass measurements of EYFP coupled to ^19^F-tags

| **Protein** | **Mass calculated [Da]** | **Mass measured [Da]** | **Intensity** | **Intensity [%]** | **Residues** |
| --- | --- | --- | --- | --- | --- |
| **EYFP-Y151AzF-tag A** | 27394.68 | 27394.73 | 255060 | 13.5 | 66222 |
| **EYFP-Y151AzF** | 27230.62 | 27230.71 | 1896078 | 100 | 67863 |
| **EYFP-Y151AzF-tag B** | 27400.65 | 27400.67 | 116284 | 20.8 | 70782 |
| **EYFP-Y151AzF** | 27230.62 | 27230.67 | 558504 | 100 | 69141 |

**Method Supplementary - Digests**

**Detailed search criteria:**

Enzyme: Trypsin or chymotrypsin, maximum missed cleavage sites: 2, N-terminus: hydrogen, C-terminus : free acid, Cys modification: Carbamidomethylation, search mode: homology search, possible multiple oxidized methionine, maximum precursor charge 3; precursor mass tolerance 10 ppm, product mass tolerance ± 0.05 Da, 1% false discovery rate. Additionally used modification, according to the modified proteins: pAzF, and modified pAzF. Data was filtered according to stringent peptide acceptance criteria, including mass deviations of ± 10 ppm, minimum 2 peptides per protein, Mascot Ion Score of at least 17 and a position rank 1 in Mascot search.

**Method Supplementary - Intact mass measurements**

**For Vmax EYFP-WT and EYFP-Y151AzF pAzFRS.2.t1:**

The protein solutions were desalted using a 0.5 mL centrifugal filter (Amicon Ultra, MWCO 3000, Millipore). A final protein concentration of 20 ng/µL in water containing 2% ACN and 0.1% formic acid (FA) was obtained. Proteins were pretreated on a nano-HPLC (Dionex Ultimate 3000) equipped with a Pepswift precolumn (monolithic, 5 x 0.2 mm) equipped with a ProSwift RP-4H column (monolithic, 100 µm x 25 cm) (Thermo Fisher Scientific) at a flow rate of 1 µL/min at 37 °C using a gradient elution (A: 0.1% FA in H_2_O, B: 0.1% FA in acetonitrile, gradient 5-60% B). Samples were measured on a maXis II ETD mass spectrometer (Bruker) with the captive spray source in positive mode with the following settings: Mass range 300-3000 m/z, 1 Hz, source voltage 1.3 kV, dry gas flow 3 L/min at 180 °C. Deconvolution of protein mass spectra was done by the MaxEnt algorithm.

The following settings were used: m/z range 10000‑50000, instrument resolving power 50000. For peak detection SNAP algorithm with following parameters was used: Quality factor threshold 0.9, S/N threshold 2 and maximum charge state of 4.
